# Activation of the G-protein coupled estrogen receptor 1 (GPER1) reduces transient receptor potential vanilloid 1 (TRPV1) activity and human iPSC-derived nociceptive neuron firing

**DOI:** 10.1186/s13287-026-05174-3

**Published:** 2026-07-07

**Authors:** Oliver Dräger, Angelique Grell, Asaria E. Vogel, Erhard Wischmeyer, Beatrice A. Nossek

**Affiliations:** Department of Cellular Neurophysiology, Medical School OWL, Bielefeld University, Bielefeld, Germany

**Keywords:** iPSC, Nociceptors, Neuronal differentiation, GPER1, TRPV1, TRESK

## Abstract

Chronic pain affects approximately one fifth of people worldwide and displays a huge burden for health care institutions. Some chronic pain conditions have a higher prevalence in women than men. Previous studies related these sex-specific differences to sex-hormone receptor activation and interaction with pain-related receptors like the transient receptor potential vanilloid 1 (TRPV1). Here, especially estrogen seems to play a crucial role. While the role of estrogen receptor alpha and beta in modulating TRPV1 has already been studied, the interplay of the membrane-bound and G-protein -coupled estrogen receptor (GPER1) with nociceptor-associated ion channels is still unknown. The study at hand focuses on the establishment of a human induced pluripotent stem cell (iPSC) -based nociceptor model to unravel the effect of GPER1 activation on nociceptor excitability. Therefore, a human dermal fibroblast cell line was successfully reprogrammed into the iPSC line BO-VC1 and subsequently differentiated into nociceptors expressing GPER1 besides TRPV1 and the Twik-related spinal cord potassium channel (TRESK). The activation of GPER1 by its specific agonist G-1 led to significantly reduced action potential firing rates and TRPV1-mediated calcium influx. In addition, GPER1 was found to downregulate TRPV1-mediated ion currents upon G-1 treatment in a human embryonic kidney cell -based model. Our findings support the hypothesis that GPER1 activation silences neuronal excitability in nociceptors, probably being a candidate for future research focusing on targeted pain modulation.

**Supplementary Information:**

The online version contains supplementary material available at 10.1186/s13287-026-05174-3.

## Introduction

Chronic pain is a global burden affecting more than 30% of people worldwide [10]. Some sorts of chronic pain are more prominent in women than men, such as migraine, rheumatological, and musculoskeletal pain [7, 71]. In this context, sex-hormones like estrogens have been reported to play an important role in sex-specific differences of chronic pain-related disease [2]. Here, the G protein-coupled estrogen receptor 1 (GPER1), a seven transmembrane-domain estrogen receptor (ER) that is involved in the activation of numerous signaling pathways under physiological and pathological conditions (reviewed in [16, 51] emerges as a prominent candidate for sex-specific differences in pain-related disorders. Besides the expression in placenta [6], lung [29], liver [75], prostate [58], ovary [28] as well as immune cells [56] it is also expressed in neuronal tissue [1] with differences in the expression levels between individuals of different age and sex [49]. Moreover, GPER1 mRNA expression is significantly higher in proliferative endometrium compared to secretory endometrium (Kolkova et al., 2010). Of note, it has also been demonstrated that GPER1 is expressed in rat trigeminal ganglion (TG) neurons that are responsible for itch and pain perception [74]. Intracellularly, in human embryonic kidney (HEK) 293 cells GPER1 is mainly expressed on the membranes of compartments like the endoplasmic reticulum or the Golgi apparatus with only little amounts being detected on the plasma membrane [57] where constitutive internalization processes take place (reviewed in [25].

In contrast to the well-studied transcription factors estrogen receptors alpha (ERα) and beta (ERβ), GPER1 mediates rapid signaling via G proteins (reviewed in [52]). In particular, GPER1 is capable to activate G proteins like Gα_s_ [67] or Gα_i_ [57] mediating rapid signaling upon binding to estradiol or specific agonists like G-1. An alternative activation of possibly Gα_q/11_ [13] and Gβ_γ_ [21] has also been discussed. Gene transcription and cell migration was reported to be affected by the presence of nuclear GPER1 after the activation by estradiol (E2) [39]. Moreover, activation of GPER1 by E2 results in downstream signaling cascades that have been shown to modulate ion channel activity like calcium (Ca^2+^)-activated potassium (K^+^) channels that are capable in mediating vasodilatation upon E2 binding in cerebral arterial myocytes [19] or the activation of epithelial sodium channels [27].

Despite GPER1, TG neurons were also shown to express the non-selective cation channel Transient-receptor potential vanilloid 1 (TRPV1) which is responsible for the transduction of noxious stimuli leading to the sensation of pain and itch in both rodents and humans [35, 53, 54, 69]. In rat TG neurons, TRPV1 contributes to the sensitization of primary headache [18]. TRPV1 can be activated by various second messengers, reactive oxygen species, ligands (reviewed in [17]) and by physical stimuli like heat [8] leading to a rapid Ca^2+^ influx and depolarization of sensory neurons with subsequent release of neuropeptides like calcitonin-gene related peptide (CGRP) [4]. Additionally, TRPV1 activity can be regulated by Ga_s_-associated receptors facilitating cAMP-dependent phosphokinase-A (PKA) modulation [3]. Moreover, TRPV1 was shown to be regulated by the Twik-related spinal cord potassium channel (TRESK) which decreases the excitability of murine dorsal root ganglion (DRG) cells and consequently acts as a modulator for pain transduction [33]. Besides the diverse regulation of TRPV1 activity and thus the excitability of sensory neurons including TGs by second messengers, a functional modulation of TRPV1 by GPER1 was previously discussed in a rodent model [35]. However, a link between the activation of GPER1 and the activity of TRPV1 or its opponent TRESK has not been elucidated on a molecular basis, as well as it was not investigated in a human model at all. In this work we investigated the role of GPER1 in regulating TRPV1 and TRESK activity in order to decode its effect on the excitability in a HEK293 and human induced pluripotent stem cell (iPSC) -derived nociceptive neuronal model.

## Methods

### Chemicals

TRPV1 inhibitor AMG517 (#HY-10634, MedChem Express, Sollentuna, Sweden), GPER1 agonist G-1 (#3577, Tocris, Bio-Techne GmbH, Wiesbaden, Germany) and GPER1 antagonist G-15 (#3678, Tocris, Bio-Techne GmbH, Wiesbaden, Germany) were dissolved in DMSO in order to get a 50 mM stock solution that were further diluted to the desired concentration.

### Molecular cloning and plasmid constructs

For the generation of the pcDNA3.1(+)_GPER1_IRES_mCherry (GPER1/mCherry) vector, the internal ribosome entry site (IRES) was amplified from pcDNA3.1_CerS4-IRES_eGFP (kindly provided by Prof. Dr. Wing-Kee Lee, University of Bielefeld, Medical Faculty, Physiology and Pathophysiology of Cells and Membranes), with Phusion® High-Fidelity DNA Polymerase (#M0530S, NEB, Ipswich, MA, USA) and specific forward primer (3’-GC*GGATCC*GATATCTAACCCCTCTCCCTCCCCCC-5’) introducing BamHI restriction site (underlined) and reverse primer (3’-GC*GAATTC*GGTTGTGGCCATATTATCATCGTGT-5’) introducing EcoRI restriction site (underlined). The amplified PCR product was purified using Monarch® PCR & DNA Cleanup Kit (#T1030S, NEB, Ipswich, MA, USA) and digested with BamHI-HF (#R3136S, NEB, Ipswich, MA, USA) and EcoRI-HF (#R3101S, NEB, Ipswich, MA, USA) restriction enzymes. Fragments were ligated into BamHI/EcoRV-digested pcDNA3.1-mCherry plasmid (#128744, Adgene [32], with T4- DNA Ligase (#M0202S, NEB, Ipswich, MA, USA) and the resulting vector was subsequently transformed into One Shot™ TOP10 chemical competent *E. coli* cells (Thermo Fisher Scientific, Darmstadt, Germany) according to manufacturer’s protocol. Next, GPER1 sequence was cut from pCMV6_GPER1 plasmid (#RC216812, Origiene, Rockville, MD, USA) using BamHI and EcoRV (#R3195S, NEB, Ipswich, MA, USA) restriction enzymes. Restriction fragments were purified by electrophoresis and gel elution using the Monarch® DNA Gel Extraction Kit (#T1020L, NEB, Ipswich, MA, USA) with subsequent ligation into BamHI/EcoRV cut pcDNA3.1(+)_IRES_mCherry plasmid with T4 DNA ligase to obtain the final pcDNA3.1(+)_GPER1_IRES_mCherry construct before transformation into chemical competent One Shot™ TOP10 *E. coli* cells.

For the generation of the pcDNA3.1_TRPV1_IRES_eGFP (TRPV1/eGFP) vector the TRPV1_IRES_eGFP sequence was cut out of the plasmid pCAGGS_TRPV1_IRES_eGFP (kindly provided by Prof. Dr. rer. nat. Thomas Voets, Laboratory of Ion Channel Research, Leuven Brain Institute, Belgium) with EcoRI and NotI (#R3189S, NEB, Ipswich, MA, USA) restriction enzymes. Restriction fragments were purified by gel electrophoresis and gel elution before ligation into EcoRI/NotI linearized pcDNA3.1(+) (kindly provided by Prof. Dr. Wing-Kee Lee, University of Bielefeld, Medical Faculty, Physiology and Pathophysiology of Cells and Membranes) vector with T4-DNA ligase. The resulting construct was transformed into One Shot™ TOP10 chemical competent *E. coli* cells (Thermo Fisher Scientific, Darmstadt, Germany). For the verification of correct insertion and amplification events, DNA sequences were determined by the CeBiTec DNA core facility, University of Bielefeld on an 3730xl Genetic Analyzer (Applied Biosystems, Weiterstadt, Germany) using BigDye-terminator v3.1 chemistry with premixed sequencing reagents (Applied Biosystems, Thermo Fisher Scientific, Darmstadt, Germany) and specific oligonucleotides for sequencing CMV-forward (5’-CGCAAATGGGCGGTAGGC-3’), F1-ori-reverse (5’-AGGGAAGAAAGCGAAAGGAG-3’), mCherry-reverse (5’- TTGGTCACCTTCAGCTTGG-3’) and IRES2-reverse (5’- CCAAAAGACGGCAATATGGTGG-3’) (Sigma-Aldrich, Merck, Darmstadt, Germany). Plasmids were isolated from *E. coli* cells for intermediate validation or transfection into mammalian cells using NucleoSpin Plasmid Mini or Midi Kits (Macherey & Nagel, Düren, Germany).

### HEK293 cell culture and transfection

Wildtype HEK293 cells and HEK293 cells stably expressing human TRESK (HEK-TRESK) (B’SYS, Witterswil, Switzerland) were cultivated in Dulbecco´s modified Eagle´s medium (DMEM; Gibco, Merck, Darmstadt, Germany) containing 10% fetal bovine serum (FBS) (Gibco, Merck, Darmstadt, Germany), 100 U penicillin/ml and 100 µg streptomycin/ml (#15140122, Thermo Fisher Scientific, Darmstadt, Germany). To maintain stable hTRESK overexpression, HEK-TRESK cells were cultivated in the presence of 15 µg/ml blasticidin (Carl Roth, Karlsruhe, Germany) and 100 µg/ml hygromycin (Carl-Roth, Karlsruhe, Germany) at 37 °C, 5% CO_2_ and 95% humidity following manufacturer’s guidelines. hTRESK expression was induced by the addition of 5 µg/ml tetracycline (Thermo Fisher Scientific, Darmstadt, Germany) (stimulation medium) for 24 h prior to analysis or transfection of HEK-hTRESK cells to the cell culture medium. All cell cultures were routinely tested for the absence of mycoplasms with the MycoGenie Rapid Mycoplasma Detection Kit (#MORV0011, Biomol, Hamburg, Germany).

To overexpress GPER1/mCherry and/or TRPV1/eGFP 0.5 × 10^6^ HEK293 or HEK-TRESK cells were seeded per six well in standard, flat base six well plates (Sarstedt, Nümbrecht, Germany) 24 h prior to transfection in cell culture medium (HEK293 cells) or stimulation medium (HEK-TRESK cells). For electrophysiological studies, cells were seeded on poly-L-lysine (Gibco, Merck, Darmstadt, Germany) coated glass coverslips. On the day of transfection, medium was changed to phenol red free DMEM medium (Gibco, Merck, Darmstadt, Germany) supplemented with 10% fetal bovine serum, charcoal stripped from USDA-approved regions (#A3382101, Gibco, Merck, Darmstadt, Germany), GlutaMAX^™^ (#35050-061, Thermo Fisher Scientific, Darmstadt, Germany) and 100 U penicillin/ml and 100 µg streptomycin/ml (Gibco, Merck, Darmstadt, Germany). Cells were transfected with a final concentration of 200 ng/ml of each plasmid: pcDNA3.1(+)_GPER1_IRES_mCherry and/or pcDNA3.1(+)_TRPV1_IRES_eGFP with pcDNA3.1(+)_mCherry and/or pcDNA3_heGFP plasmid as the respective empty vector controls. Transfection complexes were generated in OptiMEM (Gibco, Merck, Darmstadt, Germany) with Lipofectamine^™^ 2000 transfection reagent (Thermo Fisher Scientific, Darmstadt, Germany) according to the manufacturer’s protocol. 24 h after transfection cells were harvested for further analysis.

### iPSC line generation and cultivation

Reprogramming of the commercially purchased female adult human dermal fibroblasts line (HDFa; #106-05a, Cell Applications, CA, USA) into iPSCs was performed using the Epi5^™^ Episomal iPSC Reprogramming Kit (#A15960, Thermo Fisher Scientific, Darmstadt, Germany) and Lipofectamine 3000 (#L3000015, Thermo Fisher Scientific, Darmstadt, Germany) according to the manufacturer’s protocol, enabling the generation of transgene- and virus-free iPSCs. Monoclonal iPSC colonies were selected, manually picked and transferred to a new culture vessel to get rid of remaining fibroblasts and to establish a clonal iPSC culture. iPSCs were cultured on vitronectin (#A14700, Thermo Fisher Scientific, Darmstadt, Germany) coated culture vessels in Essential 8 flex medium (#A2858501, Thermo Fisher Scientific, Darmstadt, Germany) according to the manufacture’s protocol and passaged two times a week using 0.5 mM EDTA in PBS (#15575020, Thermo Fisher Scientific, Darmstadt, Germany). After expansion of the iPSC clones, cells were cryopreserved for long-term storage and further investigation in Bambanker^™^ hRM medium (#BBH01, NIPPON Genetics, Düren, Germany). Quality control of iPSCs was done by qPCR for the stem cell marker OCT4, SOX2, REX1 and NANOG (For real-time PCR Primer see Table 1; for detail protocol see RNA extraction and quantitative Real-time PCR) as well as by immunocytochemical staining for the stem cell markers OCT4, SSEA4, SOX2, TRA-1-60 and NANOG (for detailed protocol see Immunocytochemistry). Moreover, iPSCs were functionally validated by directed differentiation into all three germ layers using the StemMACS^™^ Trilineage Differentiation Kit (#130-115-660, Miltenyi Biotec, Bergisch Gladbach, Germany) according to the manufacture’s guidelines with subsequent immunocytochemical staining for meso-, endo- and ectodermal lineage markers (for detailed protocol see Immunocytochemistry). Genetic integrity of iPSCs was verified by molecular karyotyping conducted by the LIFE&BRAIN GmbH (Bonn, Germany) using Illumina Bead Array and copy number analysis, reporting copy number events if larger than 350,000 base pairs. Absence of epigenetic vectors was confirmed by RT-PCR (see RT-PCR).

### Human iPSC-derived nociceptive neuron differentiation and cell culture

Differentiation of iPSCs into nociceptive neurons was performed using a small molecule approach according to the protocol of [61] with slight modifications. Briefly, iPSCs were passaged using accutase (#17189911, Thermo Fisher Scientific, Darmstadt, Germany), 8 × 10^4^ cells were seeded on Matrigel (#CLS354277; Merck, Darmstadt, Germany) coated 6-wells in Essential 8 flex medium supplemented with 2 µM Thiazovivin (#420220, Sigma-Aldrich, Merck, Darmstadt, Germany), which was removed after 24 h. After cells reached 60–70% confluency, differentiation was started by dual SMAD inhibition with LDN-193,189 (100 nM, #04–0074, Stemgent Reprocell, Beltsville, MD, USA) and SB431542 (10 µM, #S1067, Selleckchem, Houston, TX, USA), in knockout serum replacement (KSR) medium, containing Knockout DMEM (#10829018, Thermo Fisher Scientific, Darmstadt, Germany), 15% Knock Out serum replacement (#10828010, Thermo Fisher Scientific, Darmstadt, Germany), 1% MEM Non-Essential Amino Acids (#11140050, Thermo Fisher Scientific, Darmstadt, Germany), 2 mM GlutaMAX^™^ (#35050-061, Thermo Fisher Scientific, Darmstadt, Germany), 100 U penicillin/ml and 100 µg streptomycin/ml (#15140122, Thermo Fisher Scientific, Darmstadt, Germany) and 100 µM ß-mercaptoethanol (#21985023, Thermo Fisher Scientific, Darmstadt, Germany) from day 0 to day 4. Subsequently, an overlapping inhibition of glycogen synthase kinase-3ß (GSK-3ß) with CHIR99021 (3 µM, # S1263, Selleckchem, Houston, TX, USA), vascular endothelial growth factor (VEGF) with SU5402 (10 µM, # S7667, Selleckchem, Houston, TX, USA) and Notch signaling with DAPT (10 µM, # S2215, Selleckchem, Houston, TX, USA) followed for day 2 to day 12. Additionally, KSR medium was incremented with N2/B27 medium, containing Gibco Neurobasal medium (#21103049, Thermo Fisher Scientific, Darmstadt, Germany), 1% N2 (#17502048, Thermo Fisher Scientific, Darmstadt, Germany), 2% B27 without Vitamin A (#A3353501, Thermo Fisher Scientific, Darmstadt, Germany), 2 mM GlutaMAX^™^ and 100 U penicillin/ml and 100 µg streptomycin/ml, every second day by 25% from day 4 on with medium changes every day. This led to the following gradients: Day 4–5: 25% N2/B27 and 75% KSR medium; Day 6–7: 50% N2/B27 and 50% KSR medium; Day 8–9: 75% N2/B27 and 25% KSR medium; Day 10–12: 100% N2/B27. On day 13 immature neurons were passaged using accutase and were cryoconserved in STEMdiff™ Neural Progenitor Freezing Medium (#5838, Stem cell technologies, Vancouver, BC, Canada). For final differentiation, neural progenitors were thawed and 6 × 10^4^ seeded on a poly-L-ornithine/laminin/fibronectin coated coverslip in a 24-well containing N2/B27 medium supplemented with 2 µM Thiazovivin, nerve growth factor (NGF, 25 ng/ml, # a42575, Thermo Fisher Scientific, Darmstadt, Germany), brain-derived neurotrophic factor (BDNF, 10 ng/ml, # phc7074, Thermo Fisher Scientific, Darmstadt, Germany) and glial cell line-derived neurotrophic factor (GDNF, 25 ng/ml, #450 − 10, Thermo Fisher Scientific, Darmstadt, Germany). Coating of the coverslips was done with a sitting drop of poly-L-ornithine (#P4957-50ML, Merck, Darmstadt, Germany) for 2 h at 37 °C, followed by three washing steps with PBS for 5 min and subsequent sitting drop coating with a laminin/fibronectin solution in PBS (laminin: 10 µg/ml, #CC095, Merck, Darmstadt, Germany; fibronectin: 1 µg/mL, # F1056-1MG, Sigma-Aldrich, Merck, Darmstadt, Germany) for 12 h at 4°C. On day 14, medium was changed to N2/B27 medium containing NGF, BDNF, GDNF and 4 µM 1-β-D-Arabinofuranosyl-cytosin-hydrochlorid (Ara-C, #C6645, Sigma-Aldrich, Merck, Darmstadt, Germany), which was changed on day 15 for N2/B27 medium only supplemented with the neurotrophic factors. Final maturation was done by changing the N2/B27 medium containing NGF, BDNF and GDNF every second to third day for at least 60 days.

### Reverse transcriptase -PCR of episomal vectors

iPSCs cultured in a 35 mm dish were harvested by routinely passaging as described above (iPSC generation and cultivation). As biological negative control parental HDFa cells were used and the episomal reprogramming plasmid DNA was utilized as a positive control. For genomic DNA (gDNA) isolation, cells were centrifuged for 7 min at 300xg, and cell pellets were lysed with 250–500 µl of proteinase K-lysis buffer, containing 200 mM NaCl, 20 mM Tris pH 8.5 mM EDTA pH8, 0.02% (v/v) Tween-20 and 0.5 mg/ml proteinase K (#3726.1, Carl Roth, Karlsruhe, Germany). The lysates were incubated on a shaker at 600 rpm at 55 °C for 16 h, and proteinase K was inactivated at 95 °C for 5 min. After centrifugation for 10 min at 11,000xg, the supernatant was used for PCR analysis. Quality and concentration of gDNA was assessed via Nanodrop ultraviolet spectrophotometry and adjusted to the concentration of 50 ng/µl. The PCR was performed using NEB Taq (#M0267S, New England Biolabs, Frankfurt am Mein, Germany) and specific primers for human beta actin (hACTB) as endogenous control, for EBNA-1 for the detection of all 5 episomal plasmids and for oriP that detect all but the pCXB-EBNA1 plasmid (see Table 1 for primer sequences) according to the manufacturer’s guideline. PCR was assayed using the following program: 95 °C for 90 s, 95 °C for 30 s, 58 °C for 30 s and 68 °C for 30 Sect. (30 cycles), 68 °C for 5 min in a Biometra TAdvanced Cycler (Analytik Jena, Jena, Germany). PCR samples were further analyzed by gel electrophoresis and subsequent detection with ChemiDoc MP Imaging System (Biorad, Biorad, Feldkirchen, Germany).

### RNA extraction and quantitative real-time PCR

RNA isolation was performed using NucleoSpin® RNA II Kit (Macherey & Nagel, Düren, Germany) or NucleoSpin® RNA XS Kit (Macherey & Nagel, Düren, Germany) according to the manufacturers’ guidelines. For quantitative real-time PCR analysis, cDNA was generated using the First Strand cDNA Synthesis Kit (Thermo Fisher, Germany) according to the manufacturer´s protocol. For analysis of mRNA expression levels of respective genes, a SYBR^™^ green real-time PCR assay (Applied Biosystem, Darmstadt, Germany) was performed with specific primers (Table 1).

**Table 1:** Primer sequences for the human genes analyzed by Realtime-PCR analysis

Primer	Sequence (5ʹ-3ʹ)
hGAPDH-for	ACCCACTCCTCCACCTTTGA
hGAPDH-rev	CTGTTGCTGTAGCCAAATTCGT
hACTB-for	CTTCGCGGGCGACGAT
hACTB-rev	CCACATAGGAATCCTTCTGACC
hEEF2-for	AGGTCGGTTCTACGCCTTTG
hEEF2-rev	TTCCCACAAGGCACATCCTC
hTRESK-for	GACCACACACTGGTCCTTCC
hTRESK-rev	AGATGTAGCCATAGCCCACG
hGPER1-for	CTCTTCCCCATCGGCTTTGT
hGPER1-rev	CGGGGATGGTCATCTTCTCG
hTRPV1-for	AGCTCTCCCTTCGAGTAGCA
hTRPV1-rev	CCAGTGTGCAACCAGCTAGA
hRPL36AL-for	ATCGGAAGCAGAGTGGCTATGG
hRPL36AL-rev	CAGCATCCTCTTGGATCTGCAG
hNANOG-for	GCAGAAGGCCTCAGCACCTA
hNANOG-rev	AGGTTCCCAGTCGGGTTCA
hOCT4-for	GCTCGAGAAGGATGTGGTCC
hOCT4-rev	CGTTGTGCATAGTCGCTGCT
hREX1-for	GGAATGTGGGAAAGCGTTCGT
hREX1-rev	CCGTGTGGATGCGCACGT
hSOX2-for	CACTGCCCCTCTCACACATG
hSOX2-rev	TCCCATTTCCCTCGTTTTTCT
pEP4-SF2-EBNA1	ATCGTCAAAGCTGCACACAG
pEP4-SR2-EBNA1	CCC AGG AGT CCC AGT AGTCA
pEP4-SF1-oriP	TTCCACGAGGGTAGTGAACC
pEP4-SR1-oriP	TCG GGG GTG TTA GAG ACA AC
hRPLP0-for	TGGGCAAGAACACCATGATG
hRPLP0-rev	AGTTTCTCCAGAGCTGGGTTGT
hISL1-for	GTGGAGAGGGCCAGTCTAGG
hISL1-rev	CCGTCATCTCTACCAGTTGCT
hSCN11A-for	CCTGTATGGTCAGATGAGGCTC
hSCN11A-rev	CATCACACAACCTGAGCCTGAAC
hTRKA-for	CACTAACAGCACATCTGGAGACC
hTRKA-rev	TGAGCACAAGGAGCAGCGTAGA
hBRN3A-for	CGGTAGGACTTGGCTGTGAG
hBRN3A-rev	TGTTTTCGCCCAACATGCAG

For: forward primer, rev: reverse primer

Real-time PCR reactions were conducted in triplicates in 10 µl SYBR^™^ green PCR Mastermix (Applied Biosystem, Darmstadt, Germany) in 96-well PCR plates (#21405, Starlab, Hamburg, Germany) using the following program: 50 °C for 2 min, 95 °C for 2 min, 95 °C for 15 s and 60 °C for 30 s (40 cycles) using the Analytic Jena qPCR^3^ G Tower System (Analytik Jena, Jena, Germany) and analyzed with qPCRsoft 4.1 (Analytik Jena, Jena, Germany).

### Western blotting

For western blot analysis, 1x PBS washed cells were lysed in 5x concentrated Radio-Immuno-Precipitation Assay (RIPA) lysis buffer (150 mM NaCl, 50 mM Tris-HCl, 5% Nonidet P-40 (NP-40), 2,5% sodium-deoxycholate, 0,5% SDS), 1x cOmplete™ Mini Protease Inhibitor Cocktail (Roche, Merck, Darmstadt, Germany), 5 mM EDTA pH 8.0, 1 mM PMSF) at 4 °C on a rocking shaker for 30 min and centrifuged at 10,000xg for 30 min at 4 °C. After centrifugation, the supernatant was transferred to a new reaction tube and used for determination of protein concentrations with Pierce Bicinchoninic Acid assay (BCA; #A55864, Thermo-Scientific, Oberhausen, Germany) following the manufacturer’s protocol. Protein extracts were diluted to the desired concentration in RIPA buffer, 4x Laemmli Sample Buffer (#1610747, Biorad, Feldkirchen, Germany) and β-mercaptoethanol prior to boiling at 95 °C for 5 min. Equal amounts of protein extracts were separated on a 10–12% SDS-PAGE and transferred onto polyvinylidene difluoride (PVDF) membranes using semi-dry Trans-Blot Turbo Transfer System (#1704150, Biorad, Feldkirchen, Germany) with Trans-Blot Turbo Mini 0.2 μm PVDF Transfer Packs (#1704156, Biorad, Feldkirchen, Germany) according to the manufacturer’s protocol. Membranes were blocked with blocking solution consisting of 1x Tris-buffered saline (TBST: 150 mM NaCl, 20 mM Tris-HCl and 0.1% Tween 20) and 3% low-fat milk powder or 5% bovine serum albumin (BSA)(#A7906-100G, Sigma-Aldrich, Merck, Darmstadt, Germany) at room temperature (RT) for 2 h. Primary antibodies against hACTB (1:10,000, #A5316, Mouse Monoclonal, Sigma-Aldrich, Merck, Darmstadt, Germany), Glycerinaldehyd-3-phosphat-Dehydrogenase (hGAPDH) (1:150,000, 14C10, #2118S, rabbit monoclonal, Cell Signaling Technology, Danvers, USA), hGPER1 (1:500, ab260033, rabbit monoclonal, Abcam, Cambridge, UK), hTRESK (1:8,000, # SAB3500141; rabbit polyclonal, Sigma-Aldrich, Merck, Darmstadt, Germany), and hTRPV1 (1:1,000, #SAB5700857, rabbit, polyclonal, Sigma-Aldrich, Merck, Darmstadt, Germany) were incubated in blocking solution at 4 °C overnight. Horseradish peroxidase (HRP) -conjugated species-specific secondary antibodies goat anti-rabbit IgG (#111-035-003, Jackson Immuno Research, Cambridge, UK) and goat anti-mouse IgG (#115-035-003, Jackson Immuno Research, Cambridge, UK) were used in dilutions of 1:10,000 in TBST at RT for 1 h on a tabletop shaker. HRP signals were developed by adding Western Bright Chemiluminescence Reagent (Cytiva, Buckinghamshire, UK) and detection with ChemiDoc MP Imaging System (Biorad, Feldkirchen, Germany). Data acquisition was performed using ImageLab 6.1 Software (Biorad, Feldkirchen, Germany) and data analysis was carried out using Fiji ImageJ 1.54f [60].

### Immunocytochemistry

For immunocytochemical staining of iPSCs, pre-cultured cells were passaged and seeded in a splitting ratio of 1:4 − 1:8 on vitronectin coated etched cover slips in a 24-well plate in Essential 8 flex medium as described above (iPSC generation and cultivation). At 80% confluency, the cells were fixated with 4% phosphate-buffered paraformaldehyde (# 047340.9 M; Thermo Fisher Scientific, Darmstadt, Germany) for 15 min at RT and three times washed with PBS containing 0.9 mM calcium chloride and 0.5 mM magnesium chloride (PBS^++^)). Blocking and permeabilization was performed with 1% BSA, 0.2% Triton X-100 (#108603, Sigma-Aldrich, Merck, Darmstadt, Germany) and 5% FBS in PBS^++^ for 45 min at RT for OCT4 (1:300, #ab19857, Abcam, Cambridge, UK), SSEA4 (1:200, #ab16287, Abcam, Cambridge, UK), SOX2 (1:1000, #ab97959, Abcam, Cambridge, UK) and NANOG (1:200, #ab109250, Abcam, Cambridge, UK). The iPSCs stained for TRA-1-60 (1:500, #ab16288, Abcam, Cambridge, UK) were only blocked using 1% BSA and 5% FBS in PBS^++^ for 45 min at RT. Afterwards the primary antibodies were applied and incubated for 1 h at RT. After three PBS^++^ washing steps, the secondary fluorochrome-conjugated antibodies (1:300, donkey anti-rabbit IgG Alexa 647, #A31573, Thermo Fisher Scientific, Darmstadt, Germany; 1:300, donkey anti-mouse IgG Alexa 488, #715-545-150, Jackson Immuno Research, Cambridgeshire, UK; 1:300, goat anti-mouse IgM Alexa 555, #115-165-075, Jackson Immuno Research, Cambridgeshire, UK; 1:300, donkey anti mouse IgG Cy^TM^3, #715-165-150, Jackson Immuno Research, Cambridgeshire, UK; 1:300, goat anti mouse IgG1 Alexa 488, #A21121, Thermo Fisher Scientific, Darmstadt, Germany; 1:300, goat anti guinea pig IgG Alexa 488, AB150185, Abcam, Cambridge, UK) were applied and incubated for 1 h at RT in the dark. Nuclear counterstaining was performed with 4′,6-diamidino-2-phenylindole (1 µg/mL; # D9542; Sigma-Aldrich, Merck, Darmstadt, Germany) for 10 min at RT.

Immunocytochemical staining after directed differentiation of iPSCs into all three germ layers using the StemMACS™ Trilineage Differentiation Kit was done according to the immunocytochemical staining protocol for the stem cell marker panel of iPSCs with the following slight modifications: Blocking and permeabilization was performed using 2% BSA and 0.1% Triton X-100 in PBS^++^ for 45 min at RT with subsequent overnight incubation at 4 °C with the following first antibodies: Mesodermal marker set: PDGFRB (1:100, #MA5-15143, Thermo Fisher Scientific, Darmstadt, Germany) and VE-cadherin (3 µg/ml, #MA1-198, Thermo Fisher Scientific, Darmstadt, Germany), endodermal marker set: CXCR4 (1:500, #704015, Thermo Fisher Scientific, Darmstadt, Germany) and SOX17 (1:100, #MA5-24891, Thermo Fisher Scientific, Darmstadt, Germany) and ectodermal marker set: SOX2 (1:1000, # ab97959, Abcam, Cambridge, UK) and PAX6 (1:100, # MA1-109, Thermo Fisher Scientific, Darmstadt, Germany).

Immunocytochemical staining of iPSC-derived nociceptive neurons was done according to the immunocytochemical staining protocol for the stem cell marker panel of iPSCs, too. Nevertheless, incubation with the first antibodies CGRP (1:500; # 414 004, Synaptic Systems GmbH, Göttingen, Germany), TRKA (1:500; # MA515509, Thermo Fisher Scientific, Darmstadt, Germany), GPER1 (1:500; # ab39742, Abcam, ), BRN3A (1:100, # ab245230, Abcam, Cambridge, UK), ISL1 (1:100, #H00003670-M05, Abnova, Taipeh, Taiwan), TRPV1 (1:1000, # SAB5700857, Sigma-Aldrich, Merck, Darmstadt, Germany), vGlut2 (1:400, # MAB5504, Sigma-Aldrich, Merck, Darmstadt, Germany), ßIIITUB (1:300, # PA1-41331, Thermo Fisher Scientific, Darmstadt, Germany or 1:250, #302 304, Synaptic Systems GmbH, Göttingen, Germany) and Na_V_1.9 (1:100, # ab65160, Abcam, Cambridge, UK) was done at 4 °C overnight.

Fluorescence imaging was performed using a THUNDER Imaging System with corresponding software LAS X 3.10.1.29575 and computationally deconvolution (Leica Microsystems GmbH, Wetzlar, Germany) objective HC PL APO 40x/1.30 OIL (Leica Microsystems GmbH, Wetzlar, Germany) using Leica K5 Microscope Camera (Leica Microsystems GmbH, Wetzlar, Germany). Analysis was carried out using Fiji ImageJ [60]. For quantification of the number of positive cells at least 5 pictures were analyzed.

### Flow cytometry

For the quantification of the stem cell marker expression of iPSCs, cells were cultured as described above (iPSC generation and cultivation). After harvesting, using Accutase, 1 × 10^6^ cells were stained for the surface stem cell marker TRA1-60 (1:50, #130-122-965, Miltenyi Biotec, Bergisch Gladbach, Germany) and SSEA4 (1:50, #130-124-073, Miltenyi Biotec, Bergisch Gladbach, Germany) in 25 µl PEB buffer (PBS + 0.5% BSA) for 10 min at 4 °C, washed in 500 µl PEB buffer and centrifuged at 200xg for 5 min. Subsequently, cells were incubated in inside fix solution of the Inside Stain Kit (#130-090-477, Miltenyi Biotec, Bergisch Gladbach, Germany) for 20 min at RT and centrifuged at 200xg for 5 min. After washing in 500 µl PEB buffer and centrifugation at 200xg cells were stained with the nuclear stem cell markers OCT3/4 (1:50, #130-117-821, Miltenyi Biotec, Bergisch Gladbach, Germany) and SOX2 (1:50, #130-120-790, Miltenyi Biotec, Bergisch Gladbach, Germany) in inside perm solution for 10 min at RT. Afterwards 1 ml inside perm buffer was added to the cells with subsequent centrifugation at 200xg for 5 min. Finally, cells were resuspended in 500 µl PEB buffer and analyzed by flow cytometry on a CytoFlex S (Beckman Coulter Life Sciences, Brea, CA, USA) with CytExpert 2.6 software (Beckman Coulter Life Sciences, Brea, CA, USA).

### cAMP assay

For the quantification of the intracellular cAMP concentration the HTRF cAMP Gi Detection Kit (#62IPAPEB, Revvity, Lübeck, Germany) was used with additional - HTRF IBMX Phosphodiesterase inhibitor (#62AMXADA, Revvity, Lübeck, Germany) and - HTRF forskolin adenylyl cyclase activator (#62AMYADA, Revvity, Lübeck, Germany). Assays were performed according to the manufacturers’ protocols in white ProxiPlate 384-shallow well plus plates (#6008280, Revvity, Lübeck, Germany). To test for intracellular cAMP accumulation upon GPER1 activation, G-1 or Dimethyl sulfoxide (DMSO) as vehicle control (VC) was diluted in the assay-specific stimulation buffer with equal amounts of carrier solutions being added for constant assay conditions and incubated for 1 h. HTRF fluorescence was detected at 665 nm and 620 nm using TECAN Mplex (TECAN Group, Männedorf, Switzerland).

### Calcium assay

HEK293 cells were seeded at a density of 50,000 cells per well in a clear flat base poly-L-lysine (Gibco, Merck, Darmstadt, Germany) coated 96 well plate (Sarstedt, Nümbrecht, Germany) in a volume of 150 µl and cultivated for 24 h at 37 °C, 5% CO_2_ and 95% humidity. On the day of transfection medium was changed to phenol red free DMEM medium supplemented with 10% FBS, charcoal stripped from USDA-approved regions (#A3382101, Gibco, Merck, Darmstadt, Germany), 1x GlutaMAX^™^ (#35050-061, Thermo Fisher Scientific, Darmstadt, Germany) and 100 U penicillin/ml and 100 µg streptomycin/ml (Gibco, Merck, Darmstadt, Germany). Transfections with pcDNA3.1(+)_TRPV1_IRES_eGFP or pcDNA3_heGFP were downscaled according to the protocol described above with adjustment to a plasmid concentration of 360 ng/ml. The following day, the cells were incubated with 2 µM Fluo-4-AM (Molecular Probes, Invitrogen, Eugene, OR, USA) and 0.01 vol% Pluronic F127 (Molecular Probes, Invitrogen, Eugene, OR, USA) in Hank’s buffered saline solution (HBSS) with Ca^2+^ and Mg^2+^ (#140250, Gibco, Merck, Darmstadt) containing 10 mM HEPES and 2.5 mM probenecid (Sigma-Aldrich, Merck, Darmstadt, Germany) for 30 min at 37 °C, 5% CO^2^ and 95% humidity. HBSS with Fluo-4-AM was changed to HBSS containing 1 µM G-1, TRPV1 inhibitor AMG517 or the respective amount of VC (DMSO). The plate was incubated at RT for 5 min. Fluo-4-AM fluorescence was excited at 488 nm and measured at 540 nm using TECAN Mplex (TECAN Group, Männedorf, Switzerland). After a baseline measurement of 20 s, 10x concentrated capsaicin in HBSS was added to the well using the injector to achieve a final capsaicin concentration of 10 µM per well, followed by a kinetic fluorescence measurement of 90 s. As a corresponding vehicle control ethanol was injected to a final dilution of 1:1,000. Relative fluorescence units were normalized to the baseline mean.

### Electrophysiology

Whole-cell patch-clamp recordings of HEK293 and HEK-TRESK cells were performed 24 h after transfection with respective plasmids. To record the effect of GPER1 activation on TRESK-mediated ion currents, a bath solution consisting of 135 mM NaCl, 4 mM KCl, 1.8 mM CaCl_2_, 1mM MgCl_2_, 10 mM glucose, 10 mM HEPES was prepared and pH adjusted to 7.4 with NaOH. Cells were constantly perfused with bath solution pre-heated to 37 °C. The pipette solution contained 135 mM KCl, 1mM MgCl_2_, 5 mM CaCl_2_, 5 mM Triethylene glycol diamine tetraacetic acid (EGTA), 5 mM ATP-Mg and 10 mM HEPES with pH adjusted to 7.2 with KOH (method modified after [33]). For the recording of GPER1 effects on TRPV1-mediated currents a bath solution consisting of 140 mM NaCl, 5 mM KCl, 2 mM CaCl_2_, 2 mM MgCl_2_, 10 mM HEPES) and 10 mM glucose was prepared. Cells were constantly perfused with the bath solution pre-heated to 25 °C. The pipette solution consisted of 140 mM KCl, 2 mM MgCl_2_, 5 mM EGTA and 10 mM HEPES. Both solutions were pH adjusted to 7.4 with NaOH [65]. For electrophysiological studies of iPSC-derived nociceptive neurons, cells were grown on pre-coated glass cover slips and differentiated as described above (Human iPSC-derived nociceptive neuron differentiation and cell culture). Whole-cell current- and voltage-clamp recordings were performed on day 60 of differentiation at 37 °C in a bath solution consisting of 135 mM NaCl, 5.4 mM KCl, 1.8 mM CaCl_2_, 1 mM MgCl_2_, 5 mM HEPES and 10 mM glucose. The pipette solution consisted of 120 mM potassium methanesulfonate, 4 mM NaCl, 0.5 mM CaCl_2_, 3 mM ATP-Mg, 1 mM MgCl_2_, 10 mM EGTA, 0.3 mM GTP-Tris, 10 mM HEPES and 10 mM glucose. Both solutions were adjusted to pH 7.4 with NaOH [30].

All solutions were sterile filtered with 0.22 μm (Carl-Roth, Karlsruhe, Germany) prior to use and the bath chamber was perfused with a constant flow of 0.25 ml/min with a compact perfusion system (B BRAUN, Enzersdorf, Austria). Bath solution was heated with a PTC-10/20 – Temperature Controller (npi electronic GmbH, Tamm, Germany). To analyze the effect of short term GPER1 activation on TRESK or TRPV1 currents, 5 µM G-1 or the respective VC (DMSO) were added to the bath solution during recording. TRPV1 currents were induced by the application of 10 µM (HEK cells) or 500 µM (nociceptive neurons) capsaicin diluted in the respective bath solution over a time course of 100–200 ms with a pneumatic drug ejection system PDES -DXH (npi electronic GmbH, Tamm, Germany). The TRPV1 inhibitor AMG517 was directly added to the bath solution at indicated concentrations. Patch and application pipettes were pulled from borosilicate glass capillaries (Kimble Products, UK) with a Pipette-Puller Device P-1000 (Sutter Instruments, Novato, CA, USA), and heat-polished with a Microforge CPM-2 (Scientific Instruments, Westbury, NY, USA) to give an input-resistances of 3–7 MΩ (recording pipettes) or 1.8-2.0 MΩ (application pipettes). Currents were recorded with an EPC10 (HEKA, Multi-Channel Systems MCS GmbH, Reutlingen, Germany) patch-clamp amplifier. Stimulation and data acquisition were controlled by the PatchmasterNext1.4 software package (HEKA, Multi-Channel Systems MCS GmbH, Reutlingen, Germany) and OiginPro 2024 (OriginLab Corporation, Northampton, MA, USA). Membrane currents were recorded in the whole-cell patch clamp configuration, filtered at 2 kHz with a low pass filter and digitized at 10 kHz. Series resistance was always kept below 15 MΩ and compensated at 70–80%.

### Statistical analysis

Statistical analyses were performed using GraphPad Prism 10. Data represent means ± standard error of means (SEM) of the indicated number of replicates. Normal/Gaussian distribution was tested using Shapiro-Wilk test. Results were analyzed by unpaired Student`s *t*-test, Mann-Whitney test or one-way ANOVA with either Turkey’s multiple comparison test or Dunn’s multiple comparison test. Statistical analysis of the area under curve was carried out using grouped analysis with “N” as indicated in the corresponding figure legend. Additionally, calcium assay time course recordings were analyzed using mixed group analysis with Tukey’s multiple comparisons test. Significantly different datasets were considered if p-value < 0.05 (*), p-value < 0.01 (**), p-value < 0.001 (***) or (****) p-value < 0.0001.

## Results

### GPER1 activation by G-1 reduces TRPV1-mediated ion currents in HEK293 cells

**Fig. 1:**
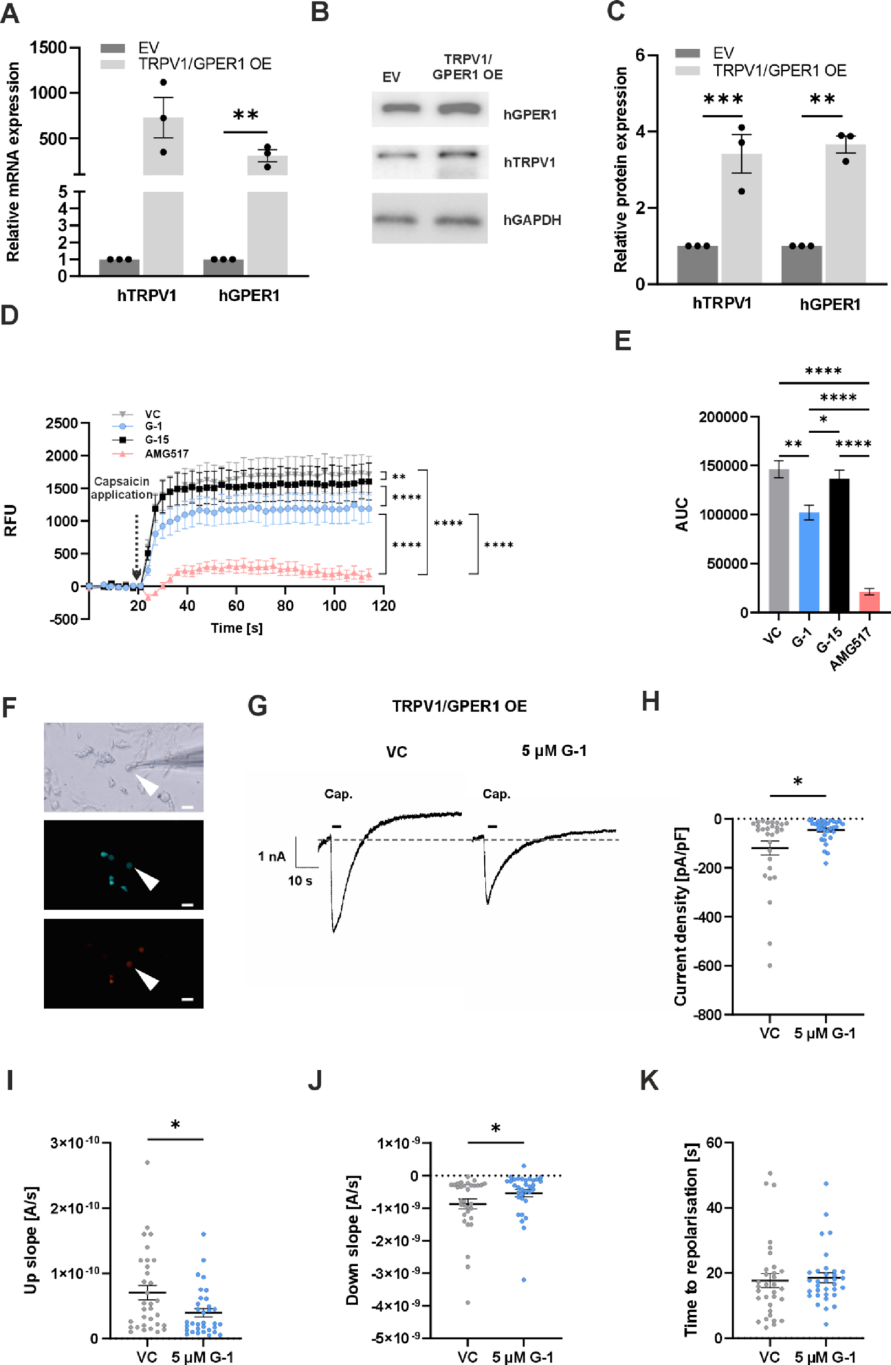
Effect of G-protein coupled estrogen receptor (GPER1) activation by specific agonist G-1 on transient receptor potential vanilloid 1 (TRPV1) - mediated ion currents. Co-overexpression of TRPV1 and GPER1 (TRPV1/GPER1 OE) in human embryonic kidney (HEK293) cells compared to empty vector (EV) control was quantified by (**A**) Realtime-PCR and (**B**, **C**) western-blot analysis (*n* = 3). Western-blot images have been cropped from the original blot as shown in additional Fig. 1. **D** Fluo-4-AM-based calcium assays revealed reduced relative fluorescence unit (RFU) values indicating reduced intracellular calcium levels after the application of 10 µM capsaicin (dotted line) in HEK293 cells overexpressing TRPV1 after the treatment with G-1, G-15 or the specific TRPV1 antagonist AMG517 compared to the respective vehicle control (VC). **E** Calculation of the area under curve (AUC) values of curves shown in (**D**) revealed significant reduction of intracellular calcium levels after the application of G-1, G-15 and AMG517 compared to VC (each *n* = 9). **F** For electrophysiological studies, exclusively green fluorescent protein (GFP) and mCherry double positive cells were chosen indicating TRPV1 (eGFP) and GPER1 (mCherry) co-overexpression (Scale bar: 200 μm). **G** Representative traces of TRPV1-mediated ion currents after the application of 10 µM capsaicin for 200 ms in the presence of G-1 or the corresponding amount of VC. Quantification of (**H**) current density, **I** up slope, **J** down slope and (**K**) time to repolarization of capsaicin-induced TRPV1 currents in TRPV1/GPER1 OE HEK293 cells in the presence of G-1 (*n* = 33) or the respective amount of VC (*n* = 32). Data were tested for normal distribution using Shapiro-Wilk test. Datapoint (n) represent independent measurements from three independent transfections. Means ± SEM (standard error of the mean) were statistically analyzed either by an unpaired (**A**,** C**) Student’s t-test, a non-parametric (**H–K**) Mann–Whitney test or (**E**) one-way ANOVA with Turkey’s multiple comparison test. (* *p* ≤ 0.05, ** *p* ≤ 0.01)

To investigate whether the activation of G-1 has an effect on TRPV1-mediated ion currents, a HEK293 cell-based GPER1/TRPV1 co-expression model was established. Therefore, HEK293 cells were transiently co-transfected with pcDNA3.1_GPER1_IRES_mCherry and pcDNA3.1_TRPV1_IRES_eGFP plasmids or the respective empty vectors (EV) as a control. The functionality of the transiently expressed TRPV1 ion channel was tested by whole-cell patch clamp measurement. The direct application of 10 µM capsaicin to the cells by a pico-applicator system led to a detectable inward directed ion current, indicating the expression of the functional TRPV1 protein being located in the plasma membrane. An overexpression of TRPV1 and GPER1 mRNA was validated by quantitative real-time PCR analysis (Fig. 1A) of co-transfected HEK293 cells relative to the EV control. Western-blot analysis further depicted a significantly increased GPER1 and TRPV1 protein expression (Fig. 1B and C).

Even though, HEK293 cells showed endogenous GPER1 expression, higher GPER1 expression levels were achieved by transient transfection. G-1, a selective GPER1 agonist, showing high binding affinity for GPER1 (K_d_=10 nM) without binding to ERα/β at concentrations as high as 10 µM (Dennis et al. 2011) was used for the selective activation of GPER1. The GPER1-overexpressing HEK293-TRESK cells showed a significant cAMP accumulation after the treatment with 5 µM G-1 for 2 h (*Additional file* 2) indicating the expression of functional GPER1.

To investigate the effect of G-1 activated GPER1 on the capsaicin-induced calcium transport into the cell, we established a plate reader -based calcium assay. After the application of 10 µM capsaicin to TRPV1-overexpressing HEK293 cells, a significant increase in Fluo-4-AM signal was detectable, indicating a TRPV1-mediated increase in intracellular calcium compared to a stimulation with the solvent of capsaicin (ethanol) alone (Additional file 3A and B). Correct Fluo-4-AM loading and performance of the assay were shown by the addition of ionomycin compared to the respective VC (*Additional file 3*,C and D). The treatment of cells with 1 µM G-1 or the TRPV1 antagonist AMG517 for 5 min and a subsequent activation of TRPV1 with capsaicin revealed a significant decrease in Fluo-4-AM signals indicating significantly lower intracellular calcium levels quantified as AUC values with 160,568 ± 9885 (VC), 102,078 ± 7472 (G-1), 136,504 ± 8834 (G-15) and 21,276 ± 3126 (AMG517) (Fig. 1D and E). Moreover, the treatment with the GPER1 antagonist G-15 showed no significant effect on TRPV1-mediated intracellular calcium accumulation compared to the VC. G-1 was also shown to significantly reduce the TRPV1-mediated intracellular calcium level compared to G-15. To further investigate the effect of G-1 on TRPV1-mediated ion currents whole-cell patch-clamp recordings were performed. For the recordings of TRPV1/GPER1 co-transfected cells exclusively mCherry and eGFP double-fluorescent cells were used (Fig. 1F) that also showed characteristic TRPV1-mediated ion currents in the presence of 5 µM G-1 or the corresponding amount of VC both supplied with the bath solution (Fig. 1G). TRPV1-ion current traces were measured by applying a membrane potential of + 60 mV while simultaneously applying 10 µM of capsaicin for 200 ms. Maximum currents were significantly reduced upon the presence of 5 µM G-1 with a mean current density of -45.6 ± 7.8 pA/pF compared to VC with a mean current density of -119.1 ± 28.8 pA/pF (Fig. 1H). Moreover, quantification of the traces downslope (Fig. 1G) upon the application of G-1 with − 5.34 × 10^− 10^±1.14 × 10^− 10^ A/s compared to VC with − 8.64 × 10^− 10^±1.52 × 10^− 10^ A/s and the upslope (Fig. 1I) of G-1 with 3.96 × 10^− 11^±6.4 × 10^− 12^ A/s compared to VC with 7.03 × 10^− 11^±1.1 × 10^− 12^ A/s (Fig. 1J) were significantly reduced indicting a reduced inward- and outward directed ion transportation velocity in the presence of G-1. The time to repolarization was not affected by the application of G-1 compared to the respective VC (Fig. 1K).

To further investigate the electrophysiological effects of GPER1 on TRESK-mediated ion currents a HEK293 cell-based GPER1/TRESK co-expression model was established. Therefore, HEK293 cells stably overexpressing functional TRESK (HEK293-TRESK) upon treatment with tetracycline were used for the transfection with pcDNA3.1_GPER1_IRES_mCherry plasmid in order to transiently overexpress GPER1 and the fluorophore mCherry. Significant GPER1 overexpression was validated by quantitative Realtime-PCR (Additional file 4A) and Western-blot (Additional file 4B and C) analysis. The expression of functional TRESK after the treatment of cells with 5 µg/ml tetracycline for 24 h was verified by whole-cell patch-clamp analysis as the treatment significantly increased TRESK-mediated ion currents (Additional file 4E and F). To analyze the effect of G-1-activated GPER1 on TRESK-mediated ion currents in the HEK293 cell model, exclusively cells that expressed mCherry were chosen indicating GPER1 overexpressing cells (Additional file 4D). There was neither a change in maximum TRESK-mediated ion currents upon activation of GPER1 after the addition of 1 µM (Additional file 4G and H) nor 5 µM G-1 (Additional file 4I and J) compared to the respective VC by delivering the agonist via the bath solution as revealed by sweep recordings from − 100 mV to 100 mV in steps of 10 mV. Hence, there was no evidence for a direct regulation of TRESK by GPER1 in our HEK293 cell-based model.

The long term treatment of HEK-TRESK cells overexpressing TRPV1 and GPER1 (Additional file 6A-C) with lower concentrations of G-1 (40 pM, 400 pM, 10 nM) for 24 h did not significantly alter the expression of these proteins compared to cells treated with the respective VC (Additional file 6D-H). Nevertheless, there was a slight decrease in TRESK protein expression upon GPER1 and TRPV1 overexpression in HEK-TRESK cells (Additional file 6B and C). Moreover, there was a slight tendency towards an increased current density after long-term treatment of GPER1/TRPV1 overexpressing HEK-TRESK cells with 10 nM G-1 compared to cells treated with the respective VC for 24 h (Additional file 6I and J). Nevertheless, there was neither a significant effect on a functional relation between GPER1 and TRESK upon the treatment with G-1, nor evidence for a long-term effect of GPER1 activation by G-1 on the translation of TRPV1 and TRESK proteins in our model.

### Induced pluripotent stem cell generation and characterization

**Fig. 2:**
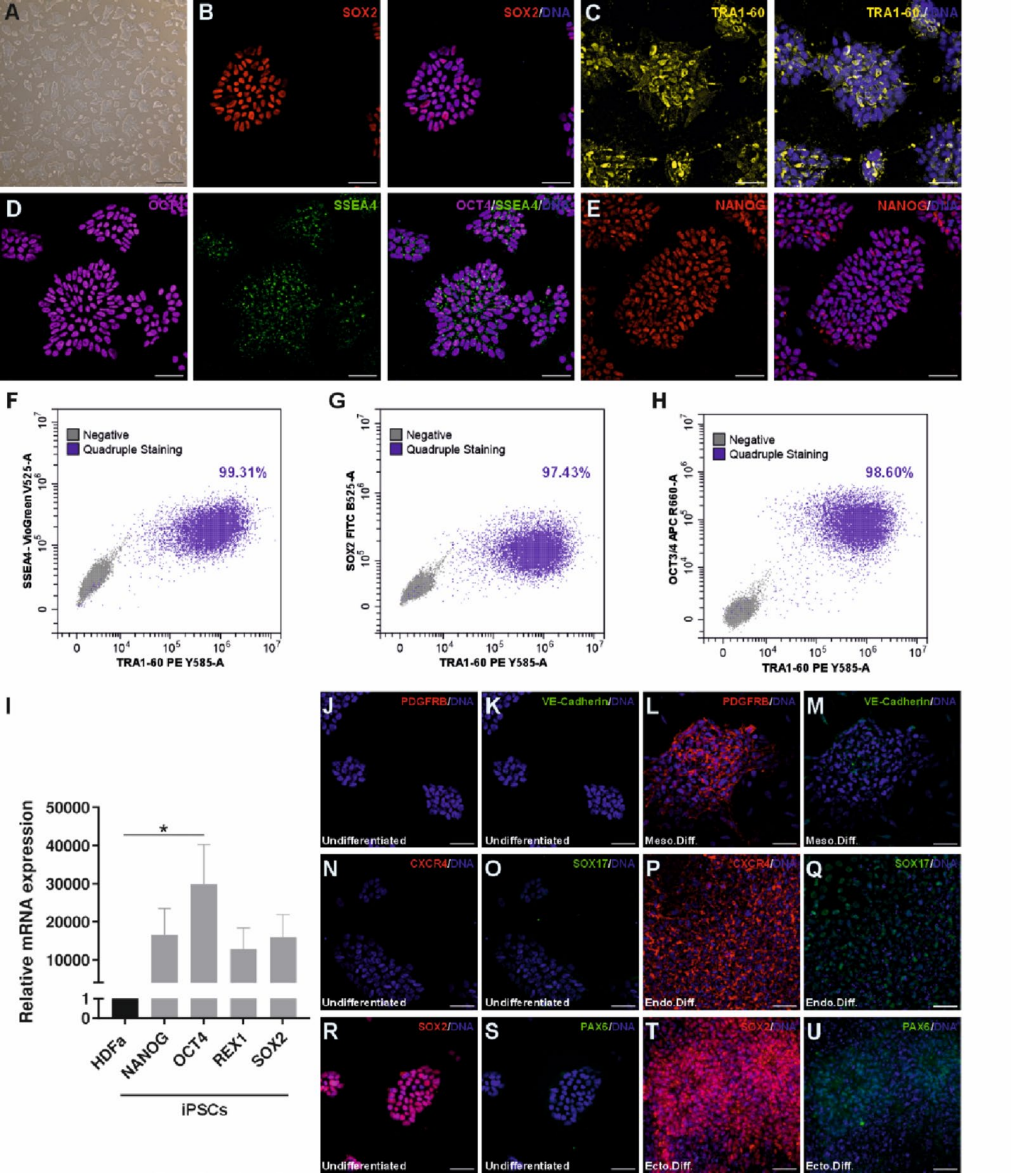
Reprogramming of adult human dermal fibroblasts (HDFa) into induced pluripotent stem cell (iPSC) line BO-VC1. Reprogramming was performed using the Epi5^™^ Episomal iPSC Reprogramming Kit, enabling the generation of (**A**) transgene- and virus-free iPSC line BO-VC1. Successful generation of fibroblast-derived iPSCs was validated by immunocytochemical staining for the pluripotency markers (**B**) SOX2, **C** TRA 1–60, **D** OCT4, SSEA4 and **E** NANOG. Quantification of the generated iPSCs via flow cytometry revealed **F** 99.31% SSEA4/TRA1-60, **G** 97.43% SOX2/TRA1-60 and **H** 98.60% OCT3/4/TRA1-60 positive cells. **I** Quantification of the transcript levels of the stem cell markers *NANOG*, *OCT4*, *REX1* and *SOX2* revealed significantly higher mRNA levels in iPSC line BO-VC1 compared to the HDFa control (*n* = 3 different passages) Moreover, generated iPSCs were functionally validated by directed differentiation into all three germ layers. Subsequent immunocytochemical staining of the iPSCs before and after differentiation confirmed the expression of (**J–M**) meso-, **N–Q** endo- and **R–U** ectodermal lineage markers only in the respective differentiations. Scale bars: 50 μm. Data were tested for normal distribution using Shapiro-Wilk test. Means ± SEM (standard error of the mean) were statistically analyzed by a Kruskal-Wallis test with Dunn’s multiple comparisons test. *n* = 3 (* *p* ≤ 0.05, ** *p* ≤ 0.01, *** *p* ≤ 0.001)

Reprogramming of HDFa cells into iPSCs was performed using the Epi5^™^ Episomal iPSC Reprogramming Kit in order to generate transgene- and virus-free iPSCs. The successful generation of the fibroblast-derived iPSC line BO-VC1 was validated by immunocytochemical staining for the pluripotency markers SOX2 (Fig. 2B), TRA 1–60 (Fig. 2C), OCT4 (Fig. 2D), SSEA4 (Fig. 2D) and NANOG (Fig. 2E). Flow cytometry analysis of the generated iPSC line BO-VC1 revealed 99.31% SSEA4/TRA1-60 (Fig. 1F), 97.43% SOX2/TRA1-60 (Fig. 2G) and 98.60% OCT3/4/TRA1-60 (Fig. 2H) positive cells following gating strategy to exclude cell aggregates (Additional file 9). Additionally, quantification of the mRNA transcript levels of the stem cell markers *NANOG*, *OCT4*, *REX1* and *SOX2* revealed elevated mRNA levels of BO-VC1 iPSC line compared to the HDFa control cells (Fig. 2I). Moreover, generated BO-VC1 iPSCs were functionally validated by trilineage differentiation into endodermal, mesodermal and ectodermal lineage. Subsequent immunocytochemical staining of the iPSC line before and after the trilineage differentiation confirmed the expression of mesodermal lineage markers Platelet-derived growth factor receptor alpha (PDGFR) and VE-cadherin (Fig. 2J-M), the endodermal lineage markers chemokine (C-X motif) receptor 4 (CXR4) and SRY-box transcription factor 17 (SOX17) (Fig. 2N-Q) and the ectodermal lineage markers paired box protein (PAX6) and SOX2 (Fig. 2R-U) in the respective differentiations. Karyotyping showed no chromosomal aberrations upon reprogramming procedure with the episomal vectors. Moreover, the absence of episomal vectors after the reprogramming procedure was validated by PCR and gel electrophoresis (Additional file 10).

### Differentiation and characterization of nociceptive neurons

**Fig. 3:**
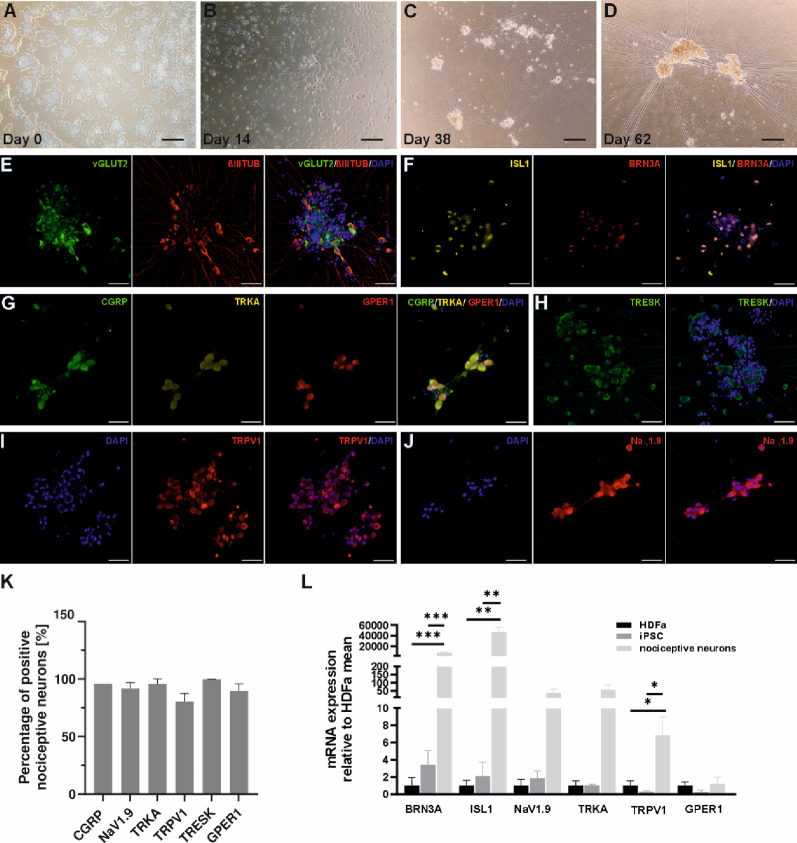
Generation of iPSC line BO-VC1 -derived nociceptive neurons. Differentiation of (**A**) human iPSC line BO-VC1 into nociceptive neurons was done by dual SMAD inhibition, suppressing TGF-ß and BMP4 signaling, in knockout serum replacement (KSR) medium (day 0–4). This was followed by an overlapping inhibition of glycogen synthase kinase-3ß (GSK-3ß), vascular endothelial growth factor (VEGF), and Notch signaling (day 2–12), with KSR medium incremented with N2/B27 medium every second day by 25% from day 4 on. After reseeding of the (**B**) immature sensory neurons, final maturation was performed in N2/B27 medium infused with the neurotrophic factors glial cell line-derived neurotrophic factor (GDNF), nerve growth factor (NGF), and brain-derived neurotrophic factor (BDNF) (day 13 - approx. 60) according to the protocols of Chambers and Schoepf leading to the differentiation of (**C**) intermediate and finally (**D**) mature nociceptors (Scale bar: 500 μm). Mature nociceptive neurons express (**E**) the neuron specific microtubule element βIII-tubulin (βIIITUB) and vGLUT2, the sensory neuron markers (**F**) insulin gene enhancer protein 1 (ISL1) as well as the brain-specific homeobox/POU domain protein 3 A (BRN3A). Additionally, iPSC-derived differentiated cells express the nociceptive markers (**G**) transient receptor potential cation channel subfamily V member 1(TRPV1), **H** voltage-gated sodium ion channel 1.9 (Na_V_1.9) and **I** TWIK-related spinal cord potassium channel (TRESK). Moreover, co-expression of (**J**) the calcitonin gene-related peptide (CGRP), the nociceptive marker high affinity nerve growth factor receptor (TRKA) and the estrogen receptor G-protein coupled estrogen receptor 1 (GPER1) was confirmed (Scale bars: 50 μm) (**K**) Quantification of the percentage of positive cells for the respective markers was 95.96 ± 2,495% for CGRP, 89.68 ± 6,137% for GPER1, 91.67 ± 5.270% for Na_V_1.9, 95.83 ± 4.167% for TRKA, 80.38 ± 7.143% for TRPV1 and 98.55 ± 0.2899% for TRESK. **L** Realtime-PCR analysis in order to quantify nociceptive neuron marker and GPER1 gene expression of HDFa-derived iPSCs (line BO-VC1) and corresponding nociceptive neurons relative to HDFa cell samples mean value. Means ± SEM (standard error of the mean) from three independent passages were statistically analyzed by one-way ANOVA with Turkey’s multiple comparison test (L). *n* = 3 (* *p* ≤ 0.05, ** *p* ≤ 0.01, *** *p* ≤ 0.001)

Human iPSC line BO-VC1 (Fig. 3A) was differentiated into nociceptive neurons by dual SMAD inhibition, suppressing TGF-ß and BMP4 signaling, in KSR medium from day 0 until day 4. This was followed by an overlapping inhibition of GSK-3ß, VEGF, and Notch signaling from day 2 until day 12 with KSR medium incremented with N2/B27 medium every second day by 25% from day 4 on. At day 12 immature sensory neurons were detached and reseeded for final maturation into nociceptive neurons. Final maturation was performed in N2/B27 medium infused with the neurotrophic factors GDNF, NGF, and BDNF from day 13 until approx. day 60 following the protocols of Chambers [9] and [61] with modification leading to the differentiation of progenitors (Fig. 3B) via intermediate (Fig. 3C) to finally mature (Fig. 3,D) nociceptors. Mature nociceptive neurons express the neuron specific microtubule element βIII-tubulin (βIIITUB) and vGLUT2 (Fig. 3E and F), the sensory neuron markers insulin gene enhancer protein 1 (ISL1) as well as the brain-specific homeobox/POU domain protein 3 A (BRN3A) (Fig. 3G and H). Additionally, 80.4 ± 7.1% of the fully differentiated cells express the nociceptive markers TRPV1 (Fig. 3G), 91.7 ± 5.3% the voltage-gated sodium ion channel 1.9 (NaV1.9) (Fig. 3H) and 98.6 ± 0.3% TRESK (Fig. 3I) giving a first indication of the functionality of the neurons. Calcitonin gene-related peptide (CGRP) was expressed by 96.0 ± 2.5% of cells, marking peptidergic nociceptiv neurons that play a crucial role in migraine (Fig. 3J). Additionally, the nociceptive marker high affinity nerve growth factor receptor (TRKA) could be detected by immunofluorescence staining in 95.8 ± 4.2% of the cells (Fig. 3J), validating the protein profile of nociceptive neurons. Moreover, immunocytochemical staining revealed an expression of the G-protein coupled estrogen receptor 1 (GPER1) (Fig. 3J) by 89.7 ± 6.1% of the nociceptive neuron population. Beides protein expression, the mRNA expression profile of the generated nociceptive neurons was quantified by Realtime-PCR analysis showing significantly higher expression of *BRN3A*, *ISL1* and *TRPV1* in nociceptive neurons compared to HDFa and the corresponding iPSC line BO-VC1. Moreover, there is a tendency towards elevated *Nav1.9*, *TRKA* and *GPER1* mRNA expression levels in nociceptive neurons compared to the respective iPSC line (Fig. 3L).

**Fig. 4:**
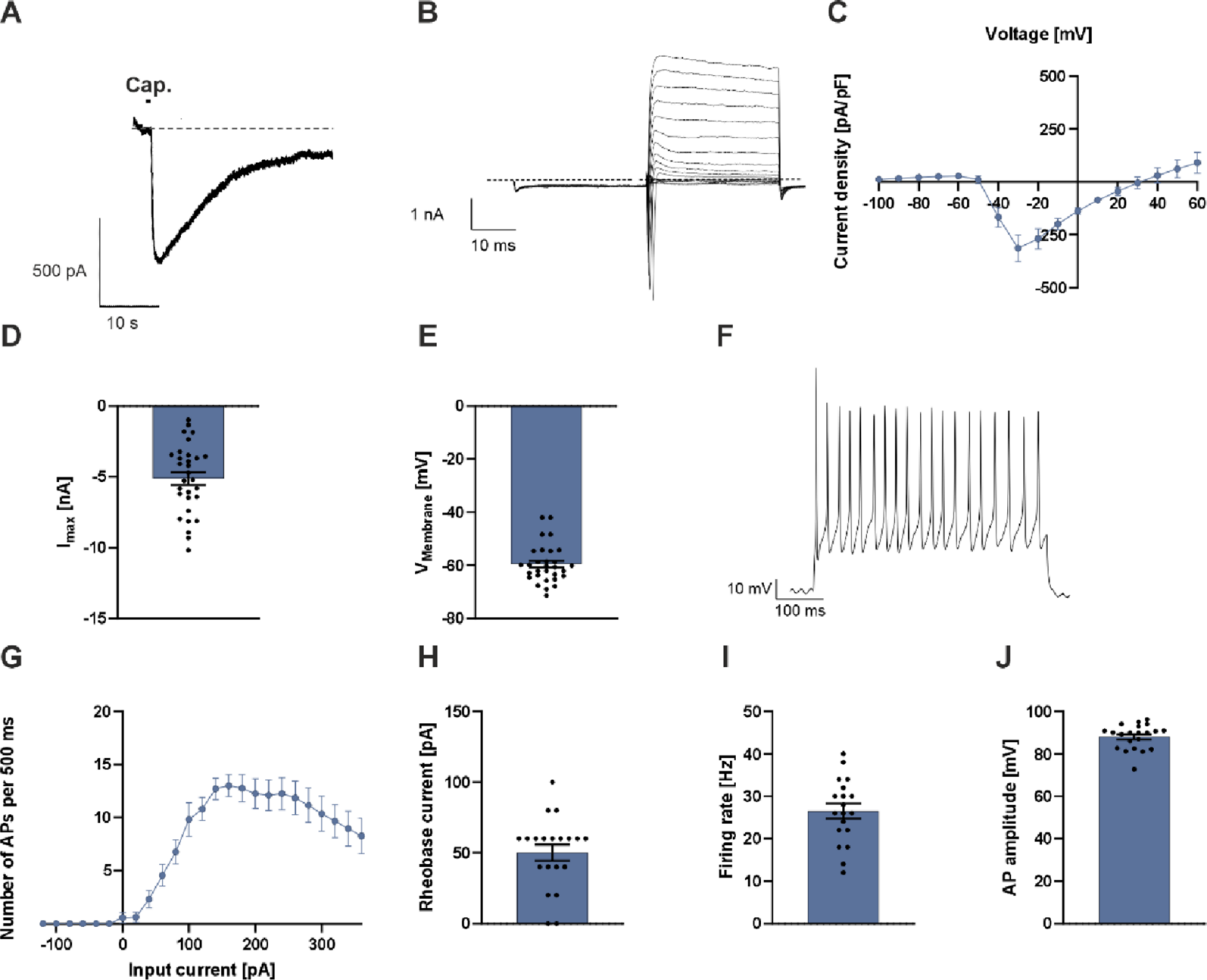
Electrophysiological properties of iPSC line BO-VC1 -derived nociceptive neurons. **A** Nociceptive neurons show characteristic TRPV1-mediated ion influx after activation with 500 µM capsaicin for 200 ms at + 60 mV holding potential. Representative sweeps of (**B**) voltage-gated sodium and potassium currents activated by voltage steps from − 120 to + 60 mV for 500 ms with an increment of 10 mV at a holding potential of − 60 mV and (**C**) IV-relation of sodium currents from nociceptive neurons (*n* = 23). **D** Quantification of maximum sodium currents of nociceptive neurons (*n* = 30) and the (**E**) resting membrane (*n* = 30) potential. **F** Trace from whole-cell current-clamp recordings of action potentials (AP) triggered by current steps from − 120 to + 360 pA for 500 ms with an increment of 20 pA showing maximum AP firing. **G** Quantification of AP numbers of nociceptive neurons (*n* = 20) for each applied current step. **H** Quantification of the rheobase current (*n* = 20), **I** firing rate of APs derived from recordings at 140 pA input current (*n* = 19), **J** AP amplitude at 140 pA input current (*n* = 21). Data shown as means ± SEM (standard error of the mean) from individually measured cells from three differentiations

Conclusively, immunofluorescence staining and quantitative Realtime-PCR analysis revealed the expression of marker mRNA and proteins that verify the ability of the adjusted protocol to generate mature nociceptive neurons. To further verify the functionality of the nociceptive neurons, whole-cell patch-clamp recordings were performed after approx. 60 days of differentiation. Functionality of TRPV1 was proven by TRPV1-mediated ion currents (Fig. 4A) upon activation by the application of 500 µM Capsaicin for 200 ms with a pico-application system and a holding potential of + 60 mV. Moreover, voltage-gated sodium and potassium currents were recorded upon activation by voltage steps from − 120 to + 60 mV for 500 ms each with an increment of 10 mV at starting from a holding potential of − 60 mV (Fig. 4B), highlighting the electrophysiological maturity of the neurons. The IV-relation of sodium currents from nociceptive neurons showed an inward directed peak current density at -30 mV holding potential (Fig. 4C) exhibiting mean maximum sodium currents with − 5.13 ± 0.45 nA (Fig. 4D) and a mean resting membrane potential of − 59.54 ± 1.33 mV (Fig. 4E). Finally, firing of action potentials (APs) triggered by current steps from − 120 to + 360 pA for 500 ms with an increment of 20 pA validated the functionality of the nociceptive neurons (Fig. 4F). Additionally, AP numbers of nociceptive neurons (Fig. 4G) for each applied current step were quantified. Cells displayed a mean rheobase current of 50.00 ± 5.71 (Fig. 4H), firing rate of APs derived from recordings at 140 pA input current with 26.53 ± 1.76 (Fig. 4I), the AP amplitude at 140 pA input current with 88.05 ± 1.27 mV (Fig. 4J).

In summary, the expression profile of neuronal and more specific nociceptive marker genes and proteins with the additional electrophysiological properties of the iPSC line BO-VC1 -derived nociceptive neurons confirmed the successful differentiation of fully mature and functional nociceptive neurons expressing TRPV1 and GPER1 as in vitro model for molecular investigations with focus on the pathophysiology of pain-related diseases.

### Activation of GPER1 by G-1 reduces the excitability of iPSC-derived nociceptive neurons

**Fig. 5:**
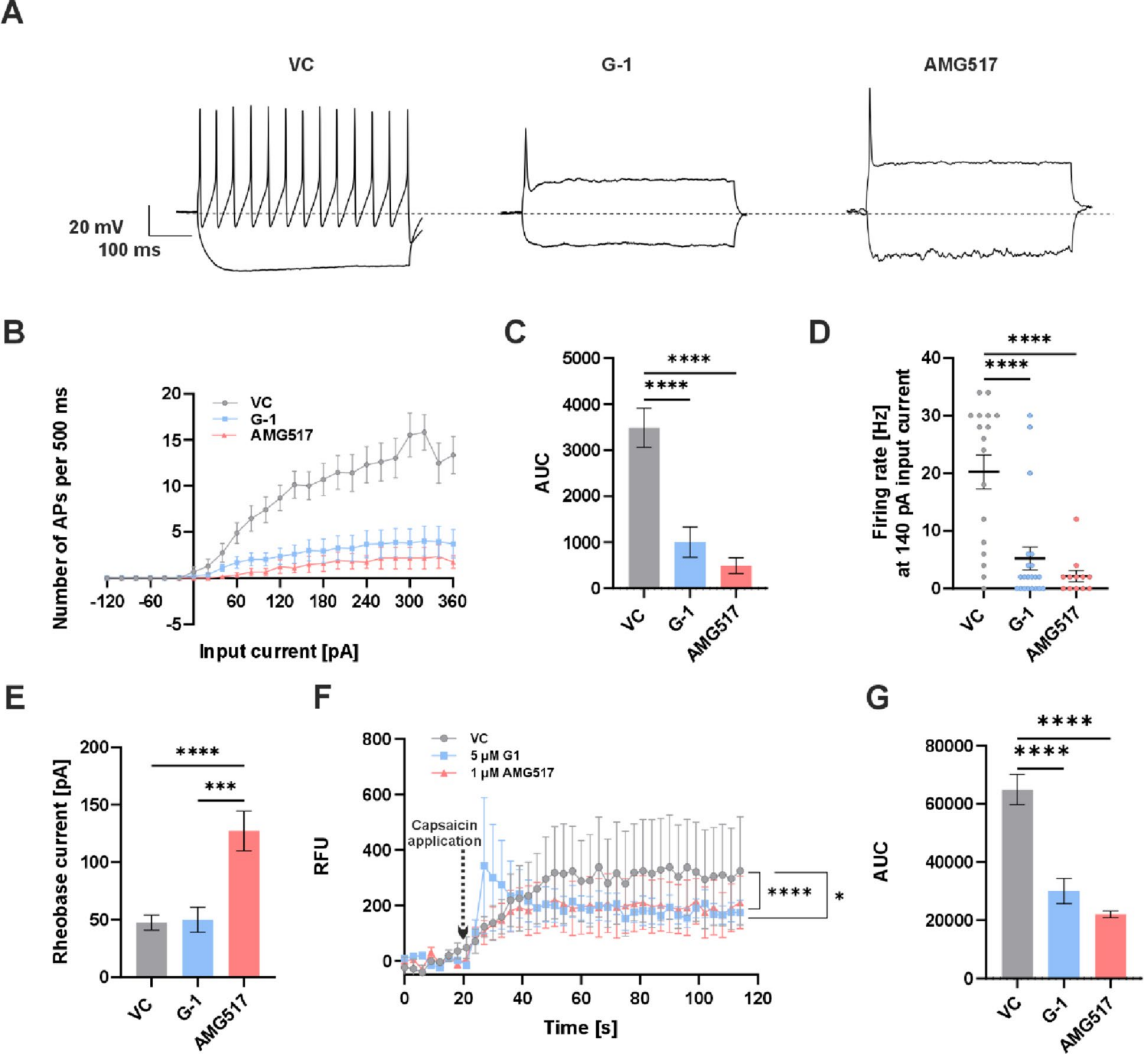
Effect of GPER1-activation by G-1 in iPSC line BO-VC1 -derived nociceptive neurons. **A** Representative current-clamp recordings of human iPSC line BO-VC1 -derived nociceptive neurons at -120 and 140 pA input current for 500 ms treated with 5 µM G-1, 1 µM TRPV1-antagonist AMG517 or the respective vehicle control (VC). **B** Quantification of action potential (AP) firing rate at indicated input currents (VC: *n* = 17; G-1: *n* = 21; AMG517: *n* = 12) per 500 ms. **C** Quantification of the area under curve (AUC) shown in (**B**) (VC: *n* = 16; G-1: *n* = 17; AMG517: *n* = 12). **D** Firing rate (VC: *n* = 17; G-1: *n* = 21; AMG517: *n* = 12) and **E** rheobase current (VC: *n* = 17; G-1: *n* = 21; AMG517: *n* = 12) of VC, G-1 or AMG517-treated nociceptive neurons. **F** Fluo-4-AM-based calcium assay time course showing relative fluorescence units (RFU). Cells were pre-treated with VC (*n* = 6), G-1 (*n* = 6) or AMG517 (*n* = 5) for 5 min prior to TRPV1 activation by the addition of 10 µM capsaicin after 20 min of baseline detection. **G** RFU was normalized to the baseline mean and analyzed by calculation of the area under curve (AUC) for statistics. Means ± SEM (standard error of the mean) were statistically analyzed by (**C**, **D**, **E**, **G**) one-way ANOVA or (**F**) mixed effect analysis with Tukey’s multiple comparisons test from individually measured cells/wells. (****p < 0.0001)

The activation of GPER1 by its specific agonist G-1 and the direct inhibition of TRPV1 by its specific antagonist AMG517 led to significantly decreased AP firing rates compared to VC in human iPSC line BO-VC1 -derived nociceptive neurons (Fig. 5A). The application of G-1 significantly reduced AP firing (Fig. 5B) with mean values of AUC (Fig. 5C) from 3486 ± 424 (VC), 1002 ± 327 (G-1) and 494 ± 170 (AMG517). Action potential firing rate decreased from 20.24 ± 2.95 Hz (VC) to 5.24 ± 1.97 Hz (G-1) and 2.17 ± 0.97 Hz (AMG517)(Fig. 5D). Additionally, the rheobase current was significantly increased upon activation by input current steps from − 120 to 360 pA with an increment of 10 pA from 47.5 ± 6.55 pA (VC) and 50 ± 10.87 (G-1) compared to 127.3 ± 17.33 pA (AMG517) (Fig. 5E). Fluo-4-AM-based calcium flux assay revealed a significant downregulation of TRPV1-mediated ion currents with an AUC for the VC with 64,955 ± 5186, G-1 with 30,055 ± 1143 and AMG517 with 22,113 ± 4358 (Fig. 5F and G).

## Discussion

The focus on sex-specific differences widely emerges in the current investigations of human diseases (reviewed in [64]. In this context, sex hormones and their respective receptors have widely been discussed as key players in pathophysiology (reviewed in [68]. Beside the long-established hormone receptors ERα and ERβ, the membrane bound estrogen receptor GPER1 was shown to play a crucial role in regulating and causing sex-specific differences in neurological and cardiovascular diseases. Of note, GPER1 is involved in signaling pathways and cellular processes that can interfere with nociception and the sensation of pain (reviewed in [16]) and was found to participate in nociception in several animal based pain models [20, 31, 36].

Nevertheless, data on GPER1’s role in nociception in human models is lacking and there is still conflicting data on whether GPER1 has an anti-nociceptive/anti-hyper analgesic effect [40] or enhances nociception [12, 43]. Novel human stem cell-derived nociceptive neuron models may therefore serve as a basis for the investigation of sensitization against nociceptive stimuli [62]. In terms of regulating nociception, the ion channel TRESK was found to modulate TRPV1 activity in murine DRG TRESK knockout cells leading to decreased excitability [33]. Moreover, there is evidence, that estrogen activates murine TRPV1 [55]. Additionally, estrogen caused phosphorylation of PKC by activation of GPER1 leading to hypersensitivity to capsaicin, acid and heat in rat DRGs [26, 41]. To investigate the interplay of GPER1 and TRPV1 in a human model, HEK293 cells were transiently transfected. Functionality of GPER1 was demonstrated by the addition of G-1 resulting in significantly increased intracellular cAMP levels indicating the activation of the G_s_ pathway [22, 44]. However, the treatment of HEK293 cells co-expressing GPER1 and TRPV1 with the GPER1 antagonist G-15 did not lead to any significant changes in intracellular calcium levels, underlining that GPER1 can be activated by the addition of G-1 in our cellular model. Fluo4-AM-based calcium assays and electrophysiological measurements validated the functionality of the TRPV1 overexpression. Interestingly, the co-expression of TRPV1 with GPER1 in HEK293 cells and subsequent treatment with G-1 led to a significant decrease in TRPV1-mediated ion currents with lower current densities, slower ion influx and efflux velocities and lower intracellular calcium levels. Previous studies showed that cAMP activates PKA that decreases desensitization of TRPV1 by direct phosphorylation at serine116 [3, 42]. This effect was also observed by the extracellular application of forskolin (FSK), an activator of the adenylyl cyclase (AC) converting ATP to cAMP. Hypothetically, cAMP activates PKA-mediated TRPV1 phosphorylation. As a consequence, this phosphorylation leads to increased TRPV1-mediated ion currents [42] which were also measurable in the here presented HEK293 model (Additional file 11). In the study at hand, the desensitization of TRPV1 by GPER1 is presumably modulated by calmodulin (CaM) via Ca^2+^-dependent activation of calcineurin [46], which needs further clarification. In this context it was shown that GPER1 induces intracellular Ca^2+^ release via its G_i_ activity and subsequent activation of the IP_3_ receptor (IP_3_R) pathway due to an activation of phospholipase C (PLC) beta1 or by direct activation of the IP_3_R [77]. Accordingly, Ca^2+^-mediated TRPV1 dephosphorylation at a capsaicin binding site was observed in rat trigeminal ganglion cells underlining the TRPV1-desensitizing role of intracellular Ca^2+^ [37].

Additionally, upon the stimulation of GPER1, the intracellular cAMP concentration was increased, underlining the binding affinity of GPER1 to G_s_ proteins [22], which was also shown in the here presented HEK293 model. Subsequent activation of downstream signaling cascades after G-protein activation, promoting AC/PKA complex formation and phosphoinositide 3-kinase (PI3K) activation [5] were shown to mediate cell survival [76], estrogen-dependent nitric oxide formation and vasodilation [24]. Additionally, G_a/q_ receptors can stimulate phospholipase-C (PKC) that leads to the dissociation of phosphatidylinositol 4,5-bisphosphate (PIP_2_) into 1,2-diacylglycerol (DAG) and (1,4,5)-inositol triphosphate (IP_3_). IP_3_ itself leads to the release of Ca^2+^ from the ER that acts as a second messenger for the activation of calcineurin/calmodulin complexes regulating TRPV1 activity (reviewed in [17], possibly playing a role also in our HEK293 model. Taken together, the observed desensitization of TRPV1 probably by the activation of GPER1 was shown to be accompanied by an increase in intracellular Ca^2+^ levels. Moreover, estrogen was shown to regulate P2 × 3 receptor -mediated peripheral pain by modulating ERα and GPER1 receptors that are expressed in primary afferent neuros [38]. So far, the direct crosstalk between GPER1 and TRESK has not been investigated. This study demonstrates that there is most likely no functional interplay between GPER1 and TRESK, as the activation of GPER1 by G-1 did not affect TRESK-mediated ion currents in the here presented HEK293 cell model. Nevertheless, the effect of G1 on endogenous TRESK expression in an iPSC-derived nociceptive neuron model needs to be investigated. Additionally, the treatment of HEK293 cells co-expressing TRESK, TRPV1 and GPER1 with physiological concentrations of G-1 did not significantly alter the expression of GPER1, TRESK or TRPV1 proteins, displaying no hint on the regulation of these proteins by GPER1 activation via G-1 on transcriptional level in a HEK293 GPER1/TRPV1 co-expression model.

To investigate the effect of GPER1 on TRPV1 in a pain associated human-based in vitro model, we established a dermal fibroblast-derived induced pluripotent stem cell line BO-VC1. iPSCs were validated according to generally accepted practice in the field [45] using FACS and qPCR analysis for pluripotency markers TRA1-60, SOX2, SSEA4, NANOG, REX1 and OCT3/4, karyotyping and three lineage differentiation. The generated iPSC line was further used for the differentiation into nociceptive neurons using the well-established Chambers protocol according to Schoepf and coworkers [9, 61] including slight modifications regarding the coating strategies and duration of differentiation. Beside the expression of the generally accepted nociceptive markers BRN3A and ISL1 [14, 61], cells also expressed the nociceptor-specific ion channels TRESK [34], Na_v_1.9 [14] (reviewed in [15]) and TRPV1 [14], with TRPV1 being also functionally validated by electrophysiology. Moreover, expression of TRKA and CGRP validated a peptidergic phenotype [34, 72] of the differentiated human nociceptors. Beside the validation of these well-established nociceptive markers, this study shows for the first time the expression of GPER1 in iPSC-derived nociceptors. The expression pattern fits the already known presence in the cell membrane and cytoplasm in most trigeminal neurons of rodents [74]. Functional characteristics of nociceptors were validated by electrophysiological recordings showing properties like action potential firing rate and sodium currents comparable to present literature [59].

The treatment of the iPSC-derived nociceptive neurons with the specific GPER1 agonist G-1 resulted in significantly reduced action potential firing rates indicating a presumably anti-nociceptive activity of GPER1 in this specific neuronal subtype and a reduction in TRPV1-mediated intracellular calcium concentration. Upon the application of capsaicin, an initial onset of the relative fluorescence in the G-1 treated cells compared to VC or AMG517 treated cells was detectable. This onset may be a first hint towards an initial pro-nociceptive activity of GPER1 by G-1 activation. Nevertheless, this initial onset and the following decay of the calcium signal must be investigated in more detail in future studies. Similar results were reported in rodents, as G-1 application reduced nociception in an α, β-methylene ATP induced pain model in vitro in DRG neurons as well as in vivo. Interestingly, the cAMP-PKA pathway was shown to play a crucial role in mediating the E2-based reduction in pain threshold, too [38], equivalently to the here presented results. Accordingly, a recent study reported that activation of GPER1 in an chronic constriction injury mouse model significantly alleviate mechanical allodynia in OVX mice [73]. More generally but still in line with the presented results, Vacca and colleagues detected reduced response to noxious stimuli after the application of estrogen on neuropathic pain and neuronal regeneration in rodents [70].

Moreover, in some studies it was shown that besides GPER1, ERα and ERβ can be activated by 17β-estradiol leading to decreased rheobase currents and a lowered threshold of action potentials facilitating spontaneous action potential firing [23]. Estrogen has been stated with both analgesic and pro-nociceptive activity depending on the animal model system, sex, age, the cellular model system, the type of pain and the underlying mechanisms [11, 16, 47]. In 2012 a study conducted by Deliu and colleagues pointed out, that GPER1 activation results in spinal nociception, involving cytosolic calcium increase, ROS accumulation, and neuronal membrane depolarization of mouse second order dorsal horn neurons [12]. Furthermore, the treatment of female mice with E2 led to significantly increased TRPV1 mRNA expression and sensitization with greater pain sensitivity of primary sensory neurons [48]. Within the here presented study, the treatment of iPSC-derived nociceptors with the TRPV1 antagonist AMG517 resulted in significantly reduced AP firing rates and calcium influx in Fluo-4-AM assays. Furthermore, a significant increase in the rheobase current and a reduced resting membrane potential (Additional file 12) was present in AMG517 treated cells compared to cells treated with the respective VC or G-1. In line with this, a knockout of TRPV1 in female mice led to hyperpolarization and thus an increased rheobase current as revealed in electrophysiological measurements of isolated hypothalamus slices [63]. Moreover, application of the TRPV1 blocker ruthenium red led to significantly reduced AP firing in this model (reviewed in [50]). Hence, the effect of GPER1 activation by G-1 in iPSC-derived nociceptive neurons is probably more likely to be due to a regulation of TRPV1-activity by intracellular signaling pathways than by direct inhibition [66], while the exact pathway needs to be investigated in future studies.

In this study, we focused on the effect of the activation of GPER1 by its specific agonist G-1 in a human iPSC-derived nociceptive neuron model to understand the role of this estrogen receptor in terms of neuronal excitability in human first-order neurons. The results highly emphasizes that GPER1 has an impact on the excitability of nociceptive neurons probably by regulating TRPV1-mediated ion currents leading to a desensitization of iPSC-derived nociceptive neurons, suggesting a putative analgesic role of GPER1 in this type of neurons. However, a direct link between GPER1 activation and the subsequent regulation of TRPV1 has to be further elucidated.

## Conclusions

This study supports the notion that estrogens display an anti-nociceptive effect by the activation of the GPER1 receptor. The interplay of GPER1 activation by its specific agonist G-1 with TRPV1 was demonstrated in a HEK293-based model. GPER1 activation could be demonstrated by intracellular cAMP accumulation and subsequent downregulation of TRPV1-mediated ion currents in whole-cell patch-clamp recordings and calcium flux assays. However, a regulating effect of GPER1 on TRESK-mediated ion currents was not detectable in our HEK293 cell-based model. Here, the direct crosstalk between GPER1 with TRPV1 or TRESK could be addressed in further studies in order to get a better insight into the regulation of the ion channels by the activation of E2 or G-1. To investigate the activation of GPER1 in a human nociceptor model, we successfully established a human dermal fibroblast-based iPSC cell line BO-VC1. This iPSC cell line is capable of being differentiated into endodermal, mesodermal and ectodermal germ layer including the differentiation into fully mature and electrophysiological active nociceptors displaying marker proteins TRPV1, Na_V_1.9, TRKA, ISL1 and BRN3A. In this human nociceptor model, GPER1 could also be detected and was shown to act in an anti-nociceptive manner by diminishing the action potential firing rate and intracellular calcium concentrations plus showing a downregulation of TRPV1-mediatd ion influx. Taken together, our data underlines the possible role of GPER1 as a down regulator for nociceptor excitability and thus being an interesting candidate for further studies focusing on targeted modulation of chronic or acute pain.

## Supplementary Information

Supplementary Material 1.

## Data Availability

All data generated or analyzed during this study are included in this published article and its supplementary information files or are accessible upon reasonable request.

## Abbreviations

AC: Adenylyl cyclase
ACTB: Beta actin
AMG517: TRPV1 inhibitor
Ara-C: 1-β-D-Arabinofuranosyl-cytosin-hydrochlorid
ßIIITUB: Beta 3 tubulin
BDNF: Brain-derived neurotrophic factor
BRN3A: Brain-specific homeobox/POU domain protein 3 A
cAMP: Cyclic adenosine monophosphate
CGRP: Calcitonin gene related peptide
CXCR4: C-X-C chemokine receptor type 4 (CXCR-4)
DRG: Dorsal root ganglion
E2: Estradiol
EEF2: Eukaryotic elongation factor 2
eGFP: Enhanced green fluorescent protein
ERα/ERβ: Estrogen receptor alpha/beta
G-1: GPER1 agonist
GAPDH: Human Glycerinaldehyd-3-phosphat-Dehydrogenase
GDNF: Glial cell line-derived neurotrophic factor
GPER1: G-protein coupled estrogen receptor 1
GSK-3ß: Glycogen synthase kinase-3ß
HDFa: (Adult) human dermal fibroblasts
HEK: Human embryonic kidney
iPSC: Induced pluripotent stem cell
IRES: Internal ribosome entry site
ISL1: Insulin gene enhancer protein
NANOG: Homeobox protein NANOG
NGF: nerve growth factor
Na_V_1.9: Voltage-gated sodium channel 1.9
OCT4: Octamer-binding transcription factor 4
PAX6: Paired box protein Pax-6
PKA: Phosphokinase A
REX1: Rex1 (Zfp-42), marker of pluripotency
RPLP36AL: Ribosomal protein L36a like
SCN11A: Sodium channel, voltage-gated, type XI, alpha subunit, also SCN11A or Nav1.9
SOX2: SRY (sex determining region Y)-box 2
SOX17: SRY-box 17
SSEA4: Stage-specific embryonic antigen-4
TG: Trigeminal ganglion
TRA-1-60: Cell surface marker for embryonic stem cells and iPSCs
TRESK: Twik-related spinal cord potassium channel
TRKA: Tropomyosin receptor kinase A
TRPV1: Transient receptor potential vanilloid 1
VEGF: Vascular endothelial growth factor
vGlut2: Vesicular glutamate transporter 2

## Declarations

### Use of generative-AI tools declaration

The authors declare that they have not used AI-generated work in this manuscript.
