# Supplementary material for: Activation of the G-protein coupled estrogen receptor 1 (GPER1) reduces transient receptor potential vanilloid 1 (TRPV1) activity and human iPSC-derived nociceptive neuron firing
Source: Stem Cell Res Ther. 2026 Jul 7;17:248. doi: 10.1186/s13287-026-05174-3 (PMC13352860; doi:10.1186/s13287-026-05174-3)
Supplement: Supplementary file 1 — Supplementary Material 1. [file 13287_2026_5174_MOESM1_ESM.docx]

**Additional files**

**
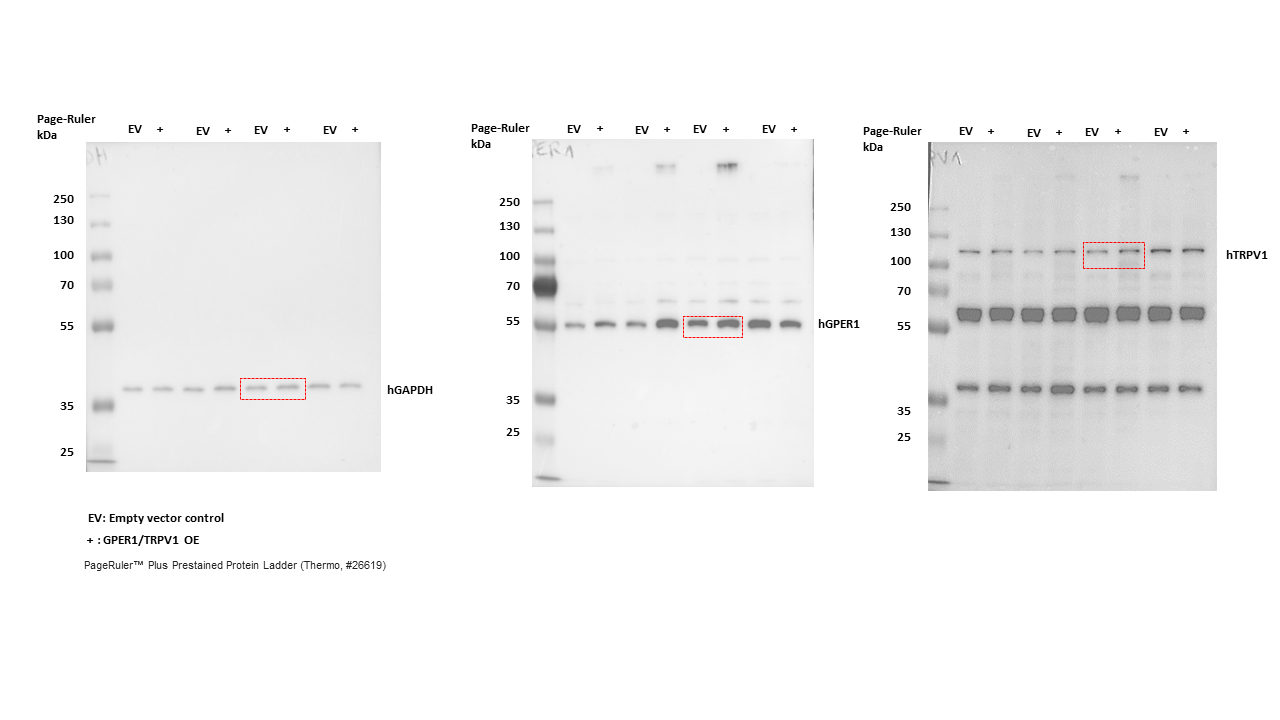
**

**Additional file 1: Western-blot raw images of GPER1 and TRPV1 overexpressing HEK293 cells.** Merge of colorimetric and chemiluminescent Western blot images for the detection of human glyceraldehyde 3-phosphate dehydrogenase (hGAPDH), G-protein coupled estrogen receptor 1 (hGPER1) and transient receptor potential vanilloid 1 (hTRPV1) channel of HEK293 cells transfected with the empty vector (EV) control or for TRPV1/GPER1 overexpression (+). Bands shown in Fig. 1 B are marked by dotted boxes.

**
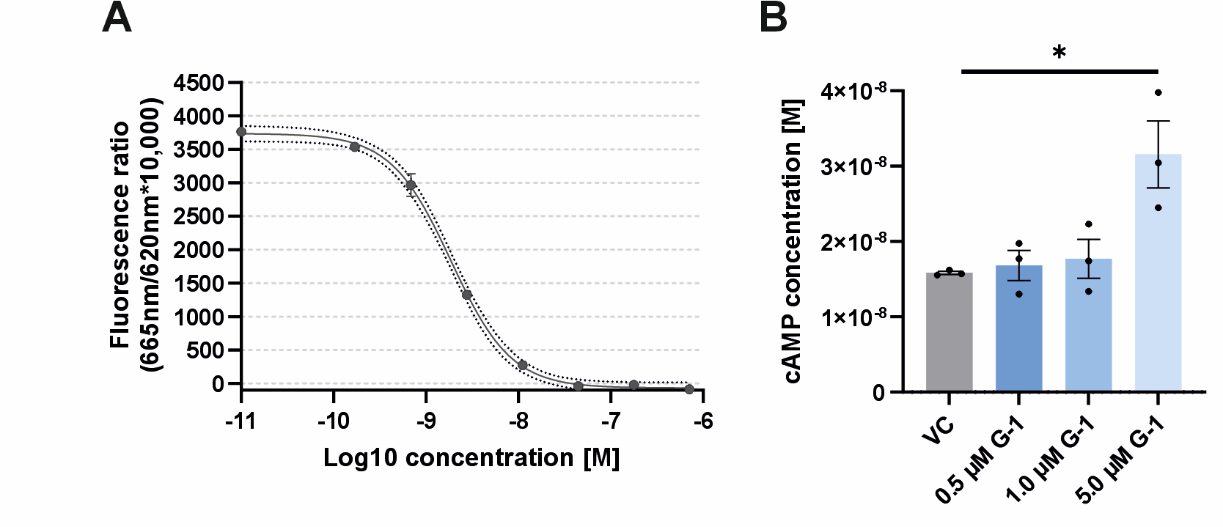
**

**Additional file 2: Validation of G protein-coupled estrogen receptor 1 (GPER1) activation by cyclic adenosine monophosphate (cAMP) assay.** HEK293 cells stably expressing TRESK (HEK-TRESK) and transiently expressing GPER1 were treated with indicated G-1 concentrations for subsequent analysis of cytosolic cAMP concentrations in comparison to cells treated with the respective vehicle control (VC). **(A)** cAMP standard curve and in relation to the standard curve calculated (n = 3 individually measured wells) **(B)** cAMP concentrations after the treatment of cells with G-1 showed a significant increase in cAMP concentration after the addition of 5 µM G-1 for 1 h (n = 3 means of individual transfections from three technical replicates). Means ± SEM (standard error of the mean) were statistically analyzed by one-way ANOVA with Turkey’s multiple comparison test. (*p ≤ 0.05)


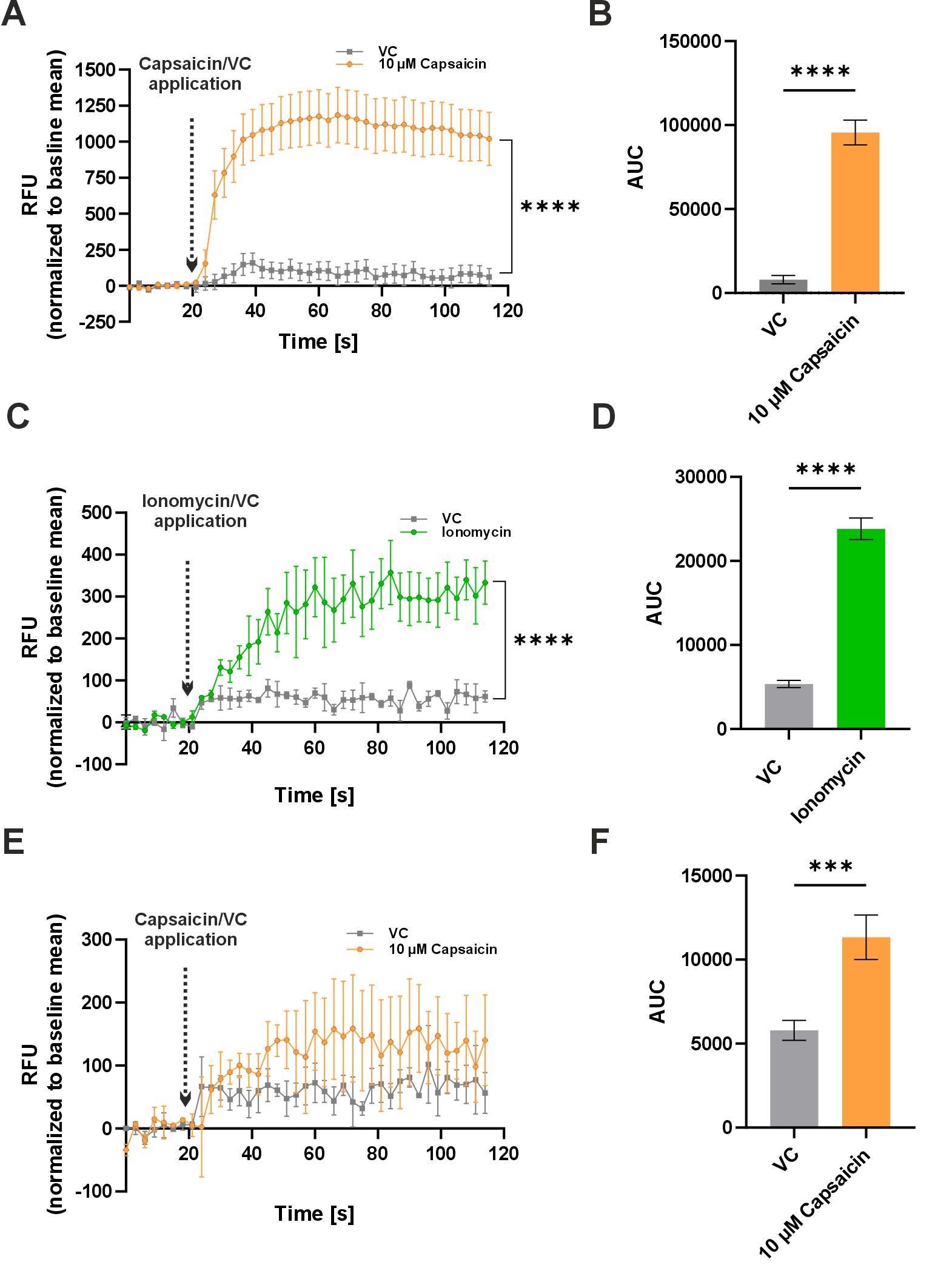


**Additional file 3: Establishment of a plate reader-based calcium assay using Fluo-4-AM. (A)** Human embryonic kidney (HEK) 293 cells were transiently transfected with the pcDNA3.1_TRPV1_IRES_eGFP vector (TRPV1) or the empty vector control pcDNA3.1_eGFP (EV). After a baseline measurement for normalization, 10 µM capsaicin or the respective vehicle control (VC) were added to the cells via an injection system (dotted line). The resulting increase in the relative fluorescence unit (RFU) was normalized to the baseline mean value of each measurement. (**B**) Calculation of the area under the curve (AUC). **(C)** Establishment of the calcium assay in iPSC-derived nociceptive neurons. Uptake of the fluo-4-AM calcium sensor into the cells was tested by the addition of Ionomycin to the cells with the injection system (dotted line) with **(D)** calculation of the area under the curve (AUC**). (E and F)** 10 µM capsaicin were sufficient to activate TRPV1-mediated ion currents compared to VC were added to the cells via an injection system (dotted line). Means ± SEM (standard error of the mean) were statistically analyzed by (A, C, E) mixed effect analysis or (B, D, F) grouped analysis via one-way ANOVA with Turkey’s multiple comparison test. (****p < 0.0001)


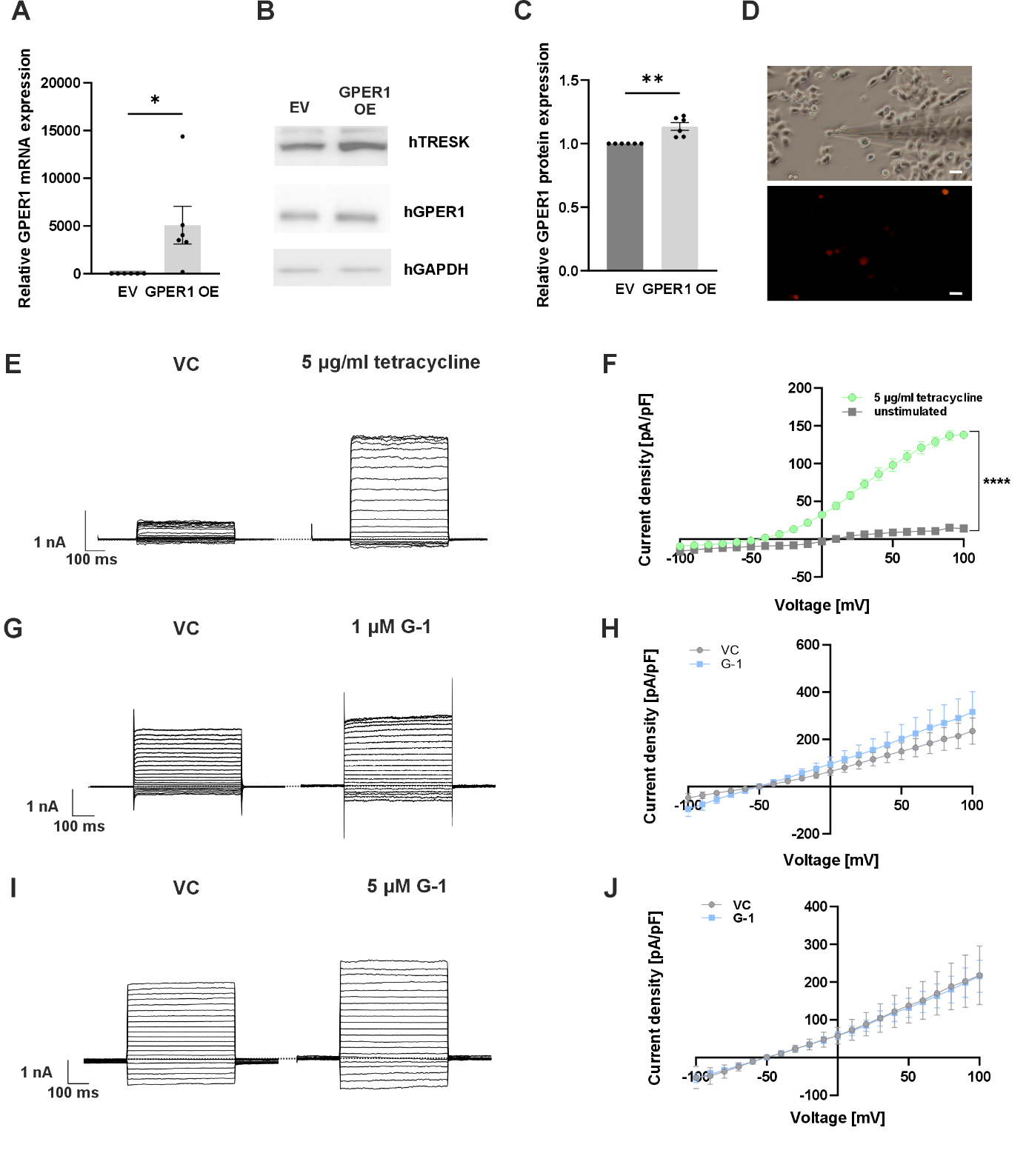


**Additional file 4:** **Effect of G-protein coupled estrogen receptor (GPER1) activation by specific agonist G-1 on the Twik-related spinal cord potassium channel (TRESK) -mediated ion currents in HEK293 cells.** Overexpression of GPER1 in human embryonic kidney cells stably overexpressing TRESK (GPER1 OE) compared to empty vector (EV) control was verified by **(A)** Realtime-PCR and **(BC)** western-blot analysis (n = 6 independent transfections). Western-blot images have been cropped from the original blot as shown in additional Fig. 5. **(D)** For electrophysiological studies mCherry-positive cells were chosen indicating GPER1 overexpression. Comparison of maximum current values obtained from current recordings showed no significant differences after the short-term application of 1 µM or 5 µM G-1 with the bath solution compared to untreated cells or the application of the vehicle control (VC) (Scale bar: 200 µm). **(E,F)** Stimulation of functional TRESK protein expression in HEK-TRESK cells upon stimulation with 5 µg/ml tetracycline for 24h leads to significantly increased outward-directed ion currents (n = 3 measured cells) in comparison to unstimulated cells (n = 3 measured cells). Means ± SEM (standard error of the mean) were statistically analyzed by an unpaired Student’s t-test of the area under curve (unstimulated: AUC = 1816 ± 140.8; 5 µg/ml tetracycline: AUC = 10311 ± 267.9). Representative sweep recordings and quantification of currents at input voltages of cells treated with **(G,H)** 1 µM G-1 (VC: n = 10; G-1: n = 16) or **(I,J)** 5 µM G-1 (VC: n = 11; G-1: n = 20) compared to cells perfused with the respective amount of VC. Data were tested for normal distribution using Shapiro-Wilk test. Means ± SEM (standard error of the mean) were statistically analyzed by an unpaired Student’s t-test (A,C,F,H and J) (* p ≤ 0.05, ** p ≤ 0.01, ****p < 0.0001)


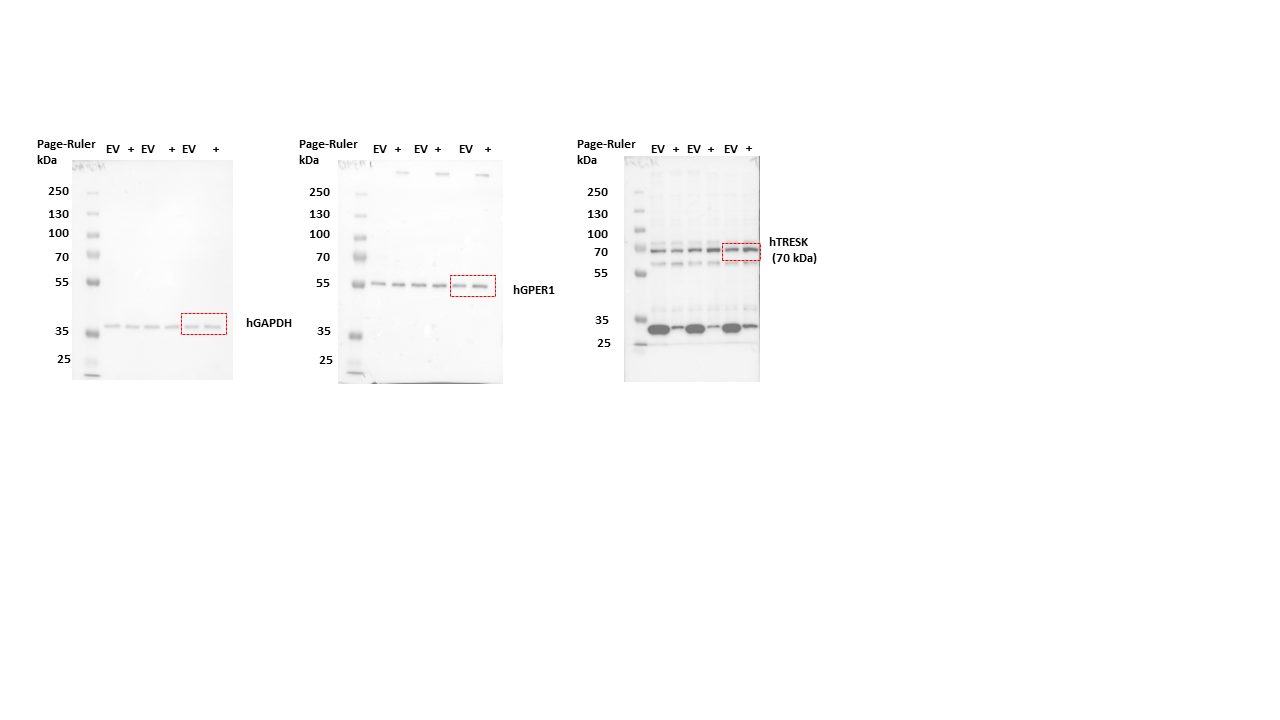


**Additional file 5: Western-blot raw images of GPER1 overexpressing HEK293 cells stably expressing TRESK (HEK-TRESK).** Merge of colorimetric and chemiluminescent Western blot images for the detection of human glyceraldehyde 3-phosphate dehydrogenase (hGAPDH), G-protein coupled estrogen receptor 1 (hGPER1) and Twik-related spinal cord potassium channel (hTRESK) of HEK293-TRESK cells transfected with the empty vector (EV) control or for hGPER1 overexpression (+). Bands shown in additional Fig. 4 B are marked by dotted boxes.


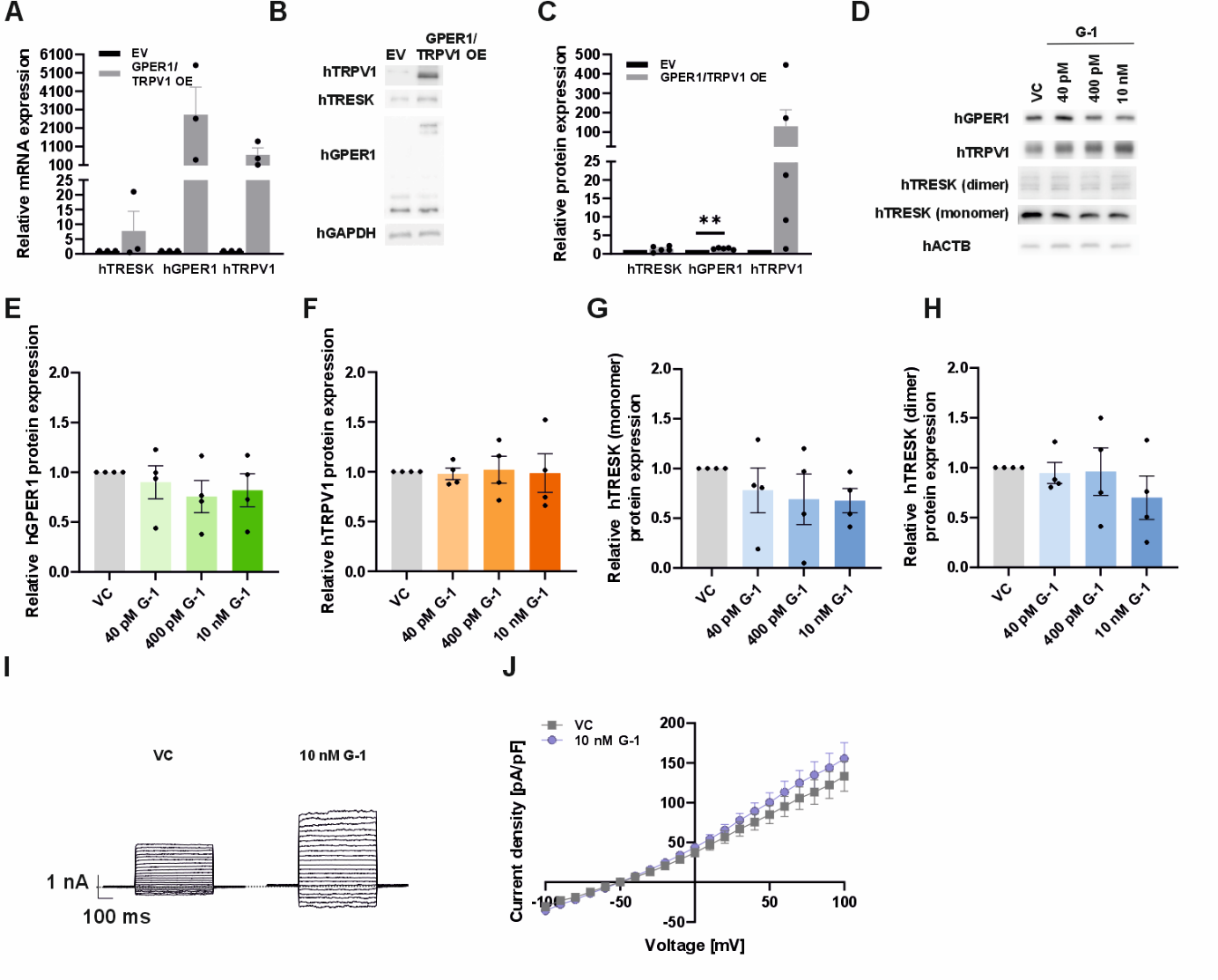


**Additional file 6: 24 h treatment of HEK293 cells co-expressing human transient receptor potential vanilloid 1 (TRPV1), Twik-related spinal cord potassium channel (TRESK) and G protein-coupled estrogen receptor (GPER1) with G-1.** HEK293 cells stably overexpressing TRESK were transfected with pcDNA3.1_GPER1_IRES_mCherry and pcDNA3.1_TRPV1_IRES_eGFP (GPER1/TRPV1 OE) or empty vector control (EV)and harvested 24 h after transfection. **(A)** Realtime-PCR analysis showed elevated TRESK, TRPV1 and GPER1 mRNA expression compared to EV. **(B)** Representative Western-blot images and **(C)** quantification of target bands reveal increased TRPV1 and GPER1 but reduced TRESK expression levels compared to EV. **(D)** Representative Western-blots of cell lysates derived from cells after 24 h treatment with indicated concentration of G-1 compared to vehicle control (VC) for TRPV1, GPER1 and TRESK 35 kDa monomer and 70 kDa dimer. Quantification of **(E)** GPER1, **(F)** TRPV1 and TRESK **(G)** mono- and **(H)** dimer expression after treatment with indicated concentrations of G-1 for 24 h compared to VC (n = 4) revealed no altered protein expression. **(I)** Typical traces and **(J)** quantification of ion currents obtained from electrophysiological recordings of HEK293 cells stably expressing TRESK after the treatment of cells with 10 nM G-1 (n = 14) or VC (n = 12) for 24 h. Western-blot images have been cropped from the original blot as shown in additional Fig. 7 (shown in B) and 8 (shown in D). Data were tested for normal distribution using Shapiro-Wilk test. Means ± SEM (standard error of the mean) statistically analyzed either by an unpaired Student’s t-test (A,C,J)or a one-way ANOVA with Turkey’s multiple comparison test (E-H). (** p ≤ 0.01)
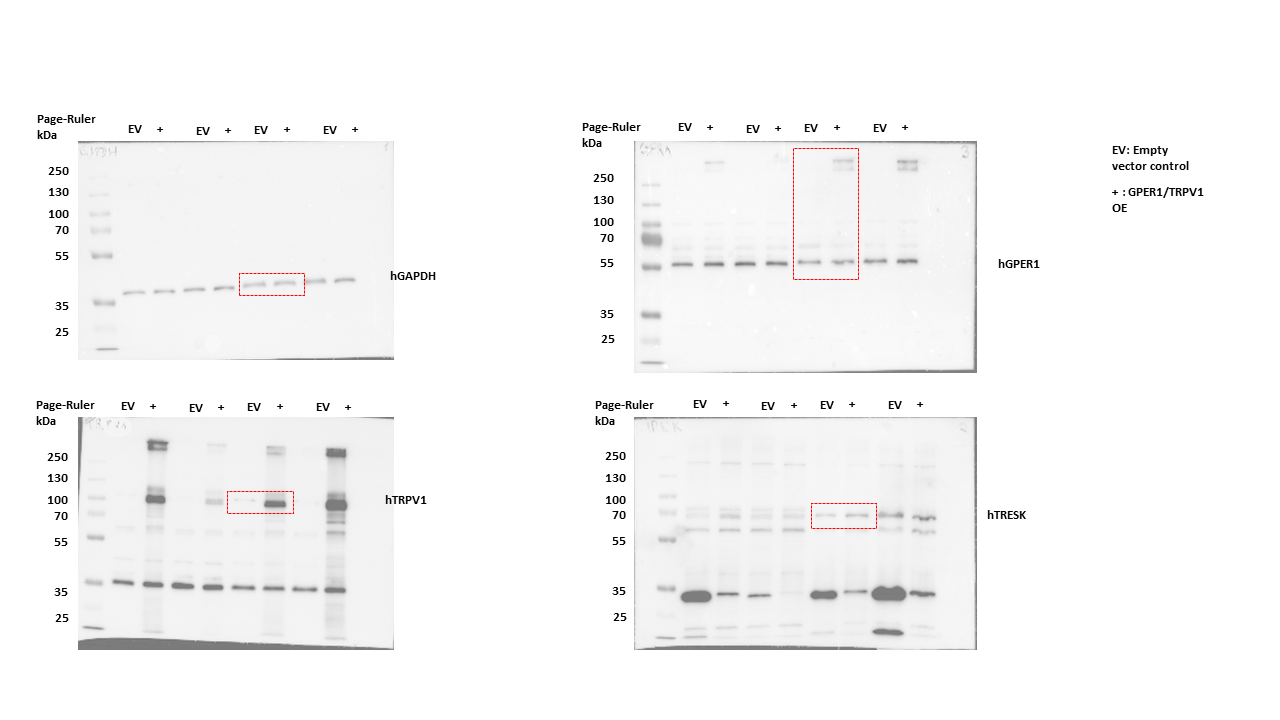


**Additional file 7: Western-blot raw images of GPER1 and TRPV1 overexpressing HEK293 cells stably expressing TRESK (HEK-TRESK).** Merge of colorimetric and chemiluminescent Western blot images for the detection of human glyceraldehyde 3-phosphate dehydrogenase (hGAPDH), G-protein coupled estrogen receptor 1 (hGPER1), the transient receptor potential vanilloid 1 (hTRPV1) receptor and Twik-related spinal cord potassium channel (hTRESK) of HEK293-TRESK cells transfected with the empty vector (EV) control or for hGPER1 overexpression (+). Bands shown in additional Fig. 6 B are marked by dotted boxes.


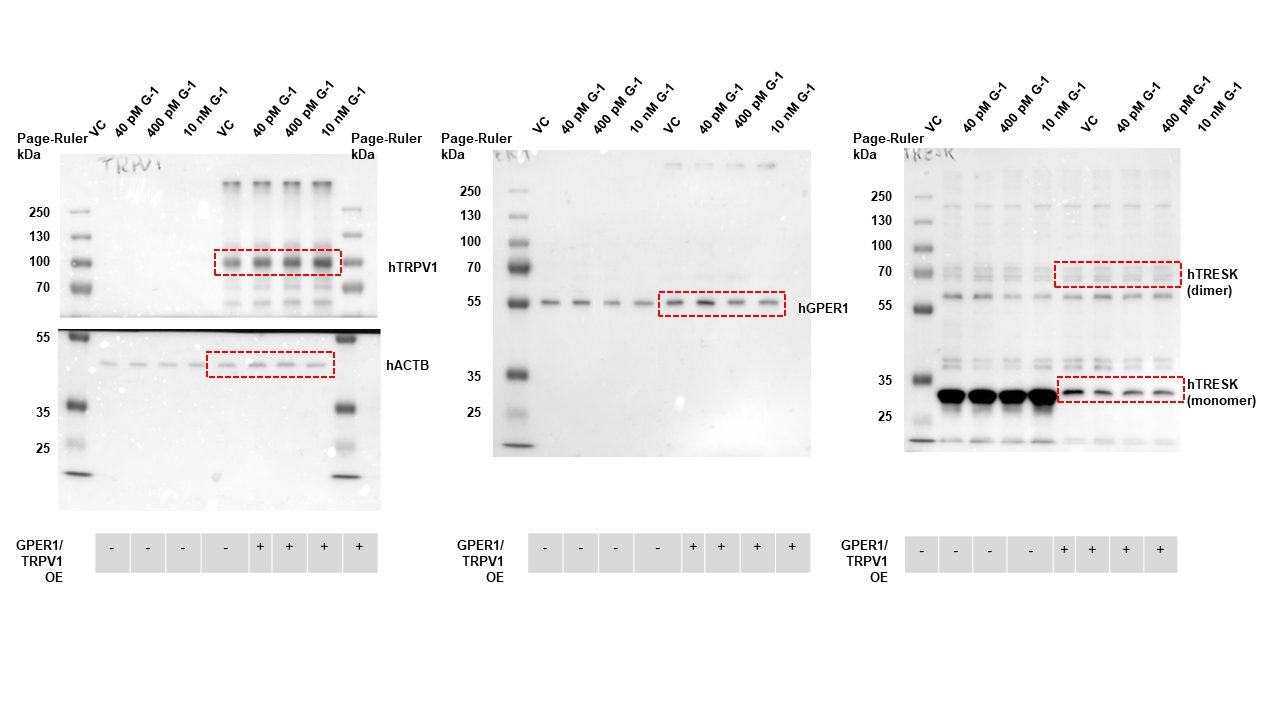


**Additional file 8: Western-blot raw images of GPER1 and TRPV1 overexpressing HEK293 cells stably expressing TRESK (HEK-TRESK) treated with G-1.** Merge of colorimetric and chemiluminescent Western blot images for the detection of human glyceraldehyde 3-phosphate dehydrogenase (hGAPDH), G-protein coupled estrogen receptor 1 (hGPER1), the transient receptor potential vanilloid 1 (hTRPV1) receptor and Twik-related spinal cord potassium channel (hTRESK) of HEK293-TRESK cells treated with indicated concentrations of the GPER1 agonist G-1. Transfected with the empty vector (-) control or for hGPER1/hTRPV1 overexpression (+). Bands shown in additional Fig. 6 D are marked by dotted boxes.

**
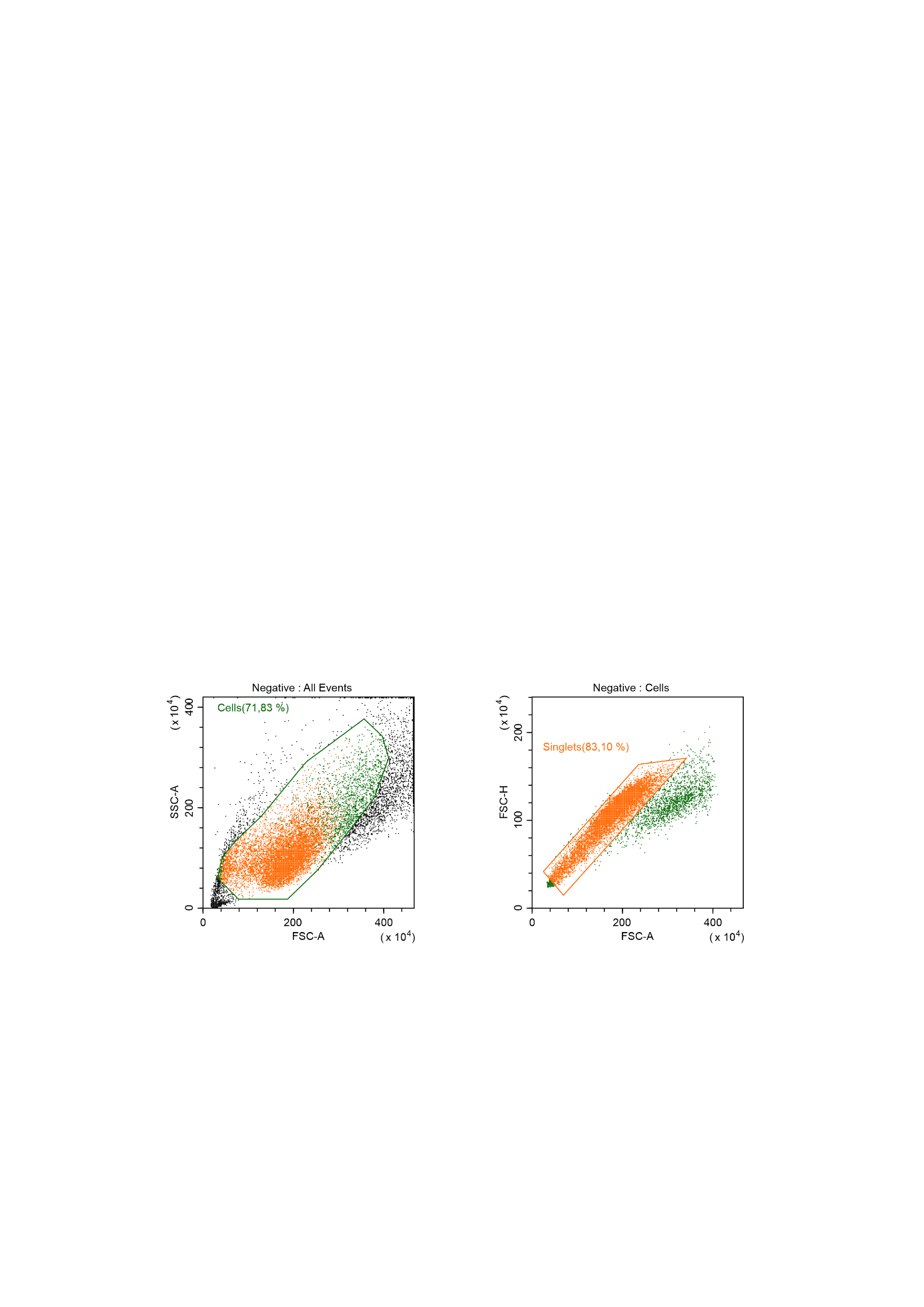
**

**Additional file 9:** **Gating strategy for cell cytometry analysis.** Cells were sorted for main population (Gate: Cells) and in a second step for single cell events (Gate: singlets) prior to analysis for positivity in respect to iPSC marker proteins.


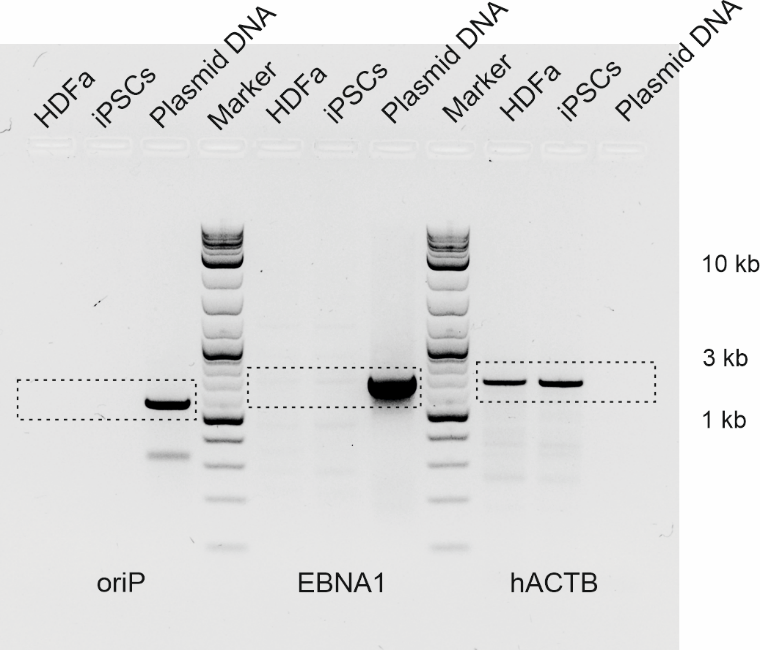


**Additional file 10: RT-PCR of episomal Vectors.** Reverse transcriptase (RT) PCR and subsequent agarose gel electrophoresis of adult human dermal fibroblast (HDFa) and induced pluripotent stem cell (iPSC) line BO-VC1 genomic DNA compared to episomal vector plasmid DNA showing absence of episomal vectors EBNA1 and oriP in the generated iPSC clone. Human beta actin (hACTB) could be detected in both cell samples.


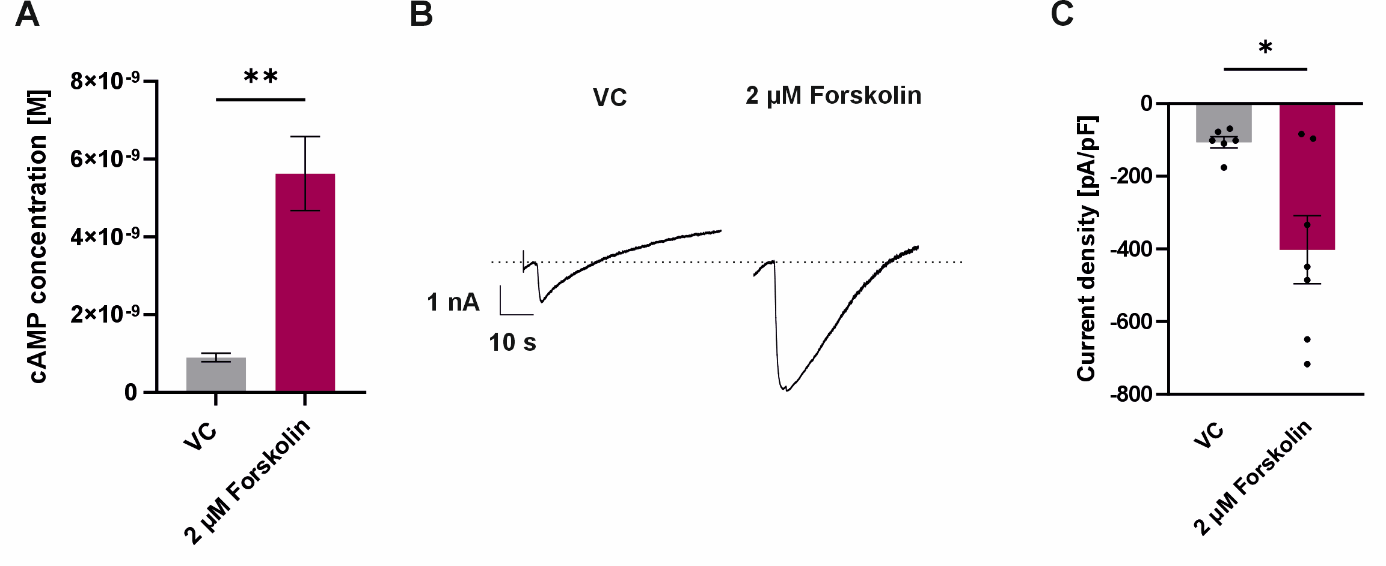


**Additional file 11: Effects of HEK293 cells overexpressing TRPV1 treated with 2 µM Forskolin on intracellular cAMP levels and TRPV1 -mediated ion currents. (A)** Treatment of cells with 2 µM Forskolin significantly increases intracellular cAMP levels as revealed by cAMP assay (VC: n = 6; Forskolin: n = 8 individually measured wells from three passages) and **(B,C)** TRPV1-mediated ion currents (VC: n = 6; Forskolin: n = 7 individually measured cells). Data were tested for normal distribution using Shapiro-Wilk test. Means ± SEM (standard error of the mean) statistically analyzed by an unpaired Student’s t-test. (*p < 0.05; ** p ≤ 0.01)

**Additional file 12: Resting membrane potential (V_m_) of iPSC-derived nociceptive neurons treated with 5 µM of the GPER1 agonist (G-1), 1 µM of the TRPV1 antagonist AMG517 or the vehicle control (VC).** Data were tested for normal distribution using Shapiro-Wilk test. Means ± SEM (standard error of the mean) statistically analyzed one-way ANOVA with Turkey’s multiple comparison test (*** p ≤ 0.001).
